# Evolution of Old World *Equus* and origin of the zebra-ass clade

**DOI:** 10.1038/s41598-021-89440-9

**Published:** 2021-05-12

**Authors:** Omar Cirilli, Luca Pandolfi, Lorenzo Rook, Raymond L. Bernor

**Affiliations:** 1 Dottorato Regionale Pegaso in Scienze della Terra, Università di Pisa, Via S. Maria 53, 56126 Pisa, Italy; 2 Dipartimento di Scienze della Terra, Paleo[Fab]Lab, Università degli Studi di Firenze, Via G. La Pira 4, 50121 Firenze, Italy; 3 Laboratory of Evolutionary Biology, Department of Anatomy, College of Medicine, 520 W St. N.W, Washington, DC 20059 USA; 4 Department of Anthropology, Human Origins Program, Smithsonian Institution, Washington, DC 20560 USA

**Keywords:** Palaeontology, Phylogenetics, Speciation, Taxonomy

## Abstract

Evolution of the genus *Equus* has been a matter of long debate with a multitude of hypotheses. Currently, there is no consensus on either the taxonomic content nor phylogeny of *Equus.* Some hypotheses segregate *Equus* species into three genera, *Plesippus*, *Allohippus* and *Equus*. Also, the evolutionary role of European Pleistocene *Equus stenonis* in the origin of the zebra-ass clade has been debated. Studies based on skull, mandible and dental morphology suggest an evolutionary relationship between North American Pliocene *E. simplicidens* and European and African Pleistocene *Equus.* In this contribution, we assess the validity of the genera *Plesippus, Allohippus* and *Equus* by cladistic analysis combined with morphological and morphometrical comparison of cranial anatomy. Our cladistic analysis, based on cranial and postcranial elements (30 taxa, 129 characters), supports the monophyly of *Equus,* denies the recognition of *Plesippus* and *Allohippus* and supports the derivation of *Equus grevyi* and members of the zebra-ass clade from European stenonine horses. We define the following evolutionary steps directly relevant to the phylogeny of extant zebras and asses: *E. simplicidens*–*E. stenonis*–*E. koobiforensis*–*E. grevyi* -zebra-ass clade. The North American Pliocene species *Equus simplicidens* represents the ancestral stock of Old World Pleistocene *Equus* and the zebra-ass clade. Our phylogenetic results uphold the most recent genomic outputs which indicate an age of 4.0–4.5 Ma for the origin and monophyly of *Equus*.

## Introduction

**Figure 1:**
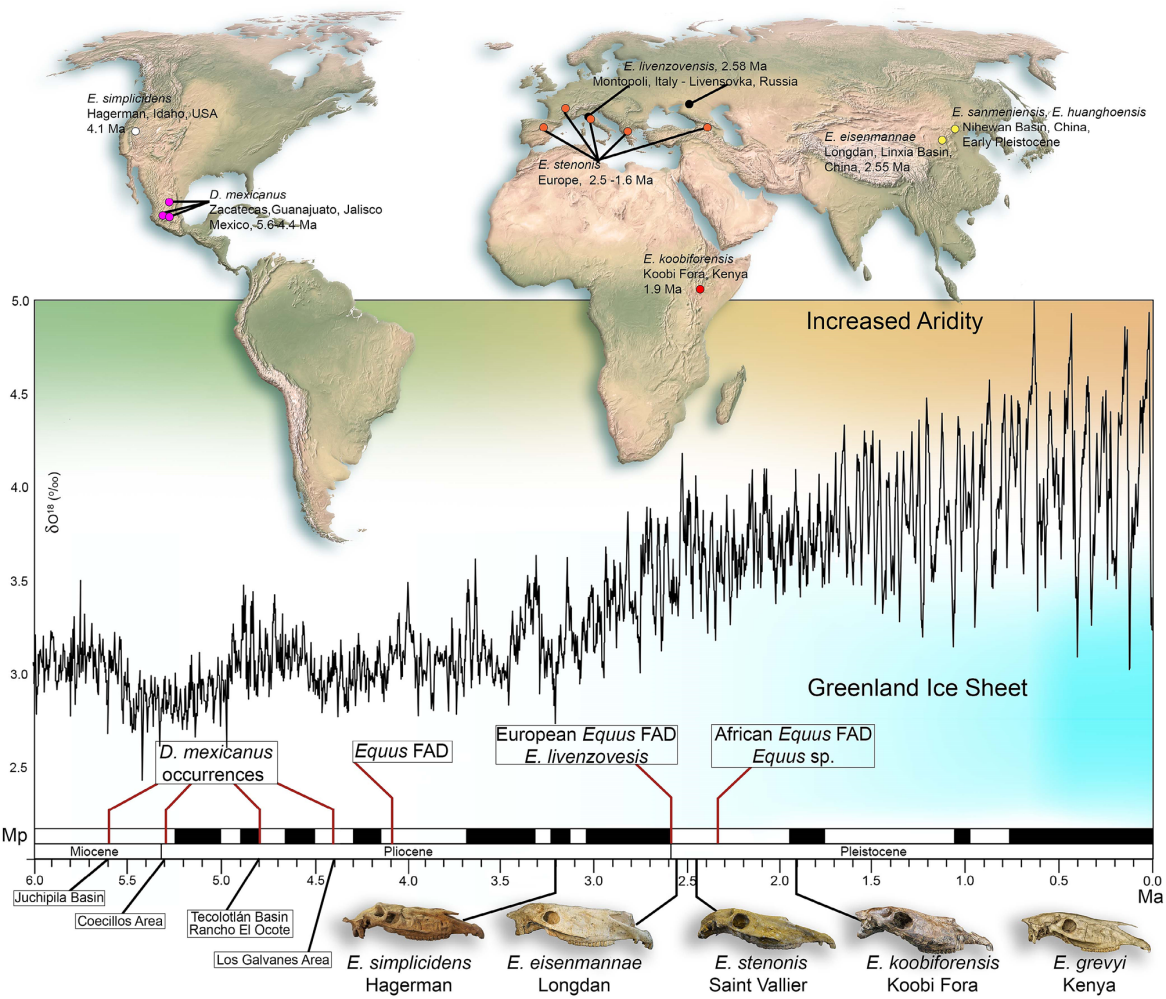
Geographic and age distribution of major events in the Old World *Equus* Evolution, including the *Equus* First Appearance Datum (FAD) in North America, 4.1 Ma^19^, the *Equus* FAD in the Old World, 2.58 Ma^17–20,22^ and the *Equus* FAD in Africa^19^. *Dinohippus mexicanus* localities are referred from recent studies^21,34^. The Old World *Equus* FAD at the base of the Pleistocene is correlated with the O^18^ isotopic global trend, marking a progressive environmental aridity since the beginning of the Pleistocene, within the paleoclimatic pulse recorded in terrestrial and marine strata, related to the initiation of a major glaciation pulse in the northern hemisphere^17–19,22,30^. Color points in map indicate the main occurrences of the selected Plio-Pleistocene fossil species (purple, *Dinohippus mexicanus*; white, *Equus simplicidens*; yellow, Chinese fossil species *Equus eisenmannae*, *Equus sanmeniensis* and *Equus huanghoensis*; black, *Equus livenzovensis*; orange, *Equus stenonis*; red, *Equus koobiforensis*). Map generated from row data of QGIS v.3.18.1 (https://www.qgis.org/it/site/) and edited by Adobe Photoshop CC2017. Abbreviations: Ma (age in million of years); Mp (Magnetic Polarity Time Scale); δ O^18^ (Ratio of stable isotope oxygen-18 and oxygen-16).

The Old World *Equus* Datum is a widely recognized biochronological event by geochronologists, correlative with the beginning of the Pleistocene, 2.58 Ma^1–22^. It is traditionally considered a significant event in the evolution of Plio-Pleistocene Old World mammalian faunas, represented by the immigration of the Pliocene North American *Equus simplicidens* into Eurasia across the Beringia land bridge^3–6,8–11,17,18,21–24^. This intercontinental dispersal is correlated with strong paleoclimatic variation documented in the terrestrial and marine records, driven by the beginning of a major glaciation pulse in the northern hemisphere^17,25^ (Fig. 1).

In the last century, evolution of the genus *Equus* was actively debated by biologists and paleontologists alike proposing a multitude of hypotheses^1–12^. Although most of the authors consider the North American *E. simplicidens* as the possible ancestor of the genus *Equus*^1–6,11,18,22^, there is no current consensus on either the taxonomic content nor phylogeny of *Equus*. In fact, *Equus’* traditional taxonomy was upended when some investigators proposed segregating the genus into three genera^7^: North American Pliocene *Plesippus*^13^ (type species *E. simplicidens*), Pleistocene Eurasian and African *Allohippus*^14^ (type species *E. stenonis*) older than 1 Ma, and previously recognized species of *Equus* less than 1 Ma as being the sole members of the genus. The segregation into these three genera was based on cranial morphology and proportion^7^. Notably, some studies used ten metric characters to distinguish Plio-Pleistocene species and extant *Equus*^2^, whilst more recent studies used only a single character (size of the cranium, brain-box) to distinguish among *Plesippus*, *Allohippus* and *Equus*^7^. The morphology of the dentition and postcranial elements was never taken into account. The validity of North American *Plesippus* and the European *Allohippus* was supported by a recent morphological qualitative cladistic analysis^12^, whereas another cladistic study supported the hypothesis of *E. simplicidens* as possible common ancestor for species of the genus *Equus*^20^. During the last decades, the Chinese species *Equus E. qingyangensis* was included within the genus *Plesippus* (*P. qingyangensis*)^26^, whereas the Chinese species *Equus sanmeniensis* (*Allohippus sanmeniensis*)^27^, the European Early Pleistocene species *Equus livenzovensis* and *Equus senezensis* (*Allohippus livenzovensis* and *Allohippus senezensis*)^27–29^, and the African Early Pleistocene *Equus koobiforensis* (*Allohippus koobiforensis*)^29^ were assigned to the genus *Allohippus*. Furthermore, other authors ^27^, regarded *Allohippus* as a subgenus of *Plesippus* for the European Early Pleistocene species *Plesippus* (*Allohippus*) *livenzovensis* and *Plesippus* (*Allohippus*) *stenonis*.

Another controversial issue concerns European Pleistocene *Equus stenonis* role in the evolutionary history of *Equus* and the origin of the zebra-ass clade. Early studies^2,15,16^ suggested a relationship between *Equus stenonis* and extant *Equus grevyi* based on skull and dental morphology. A morphological similarity was further identified in the skull and dentition between *E. stenonis*, *E. koobiforensis* (Kenya, Africa, 1.9 Ma) and *E. grevyi*, suggesting that *E. koobiforensis* could be more closely related to European *E. stenonis* than the Chinese *E. sanmeniensis*^2^. Furthermore, some similarities were highlighted in skull, mandible and dental morphology between *E. stenonis* and *E. simplicidens*, suggesting that *E. stenonis* exhibits an intermediate morphology between the North American *E. simplicidens* and the African *E. koobiforensis*^2^. Nevertheless, other hypotheses^3^ identified *E. stenonis* as a branch of the *E. simplicidens*–*E. sanmeniensis*–*E. koobiforensis*–*E. grevyi* evolutionary lineage.

Herein, we assess the validity of the genera *Plesippus, Allohippus* and *Equus* by means of our cladistic analysis*.* We define the evolutionary relationships of *E. stenonis* to other Old World Pleistocene and extant *Equus* and the origin of the zebra-ass clade.

## Results

### Phylogenetic analysis

**Figure 2:**
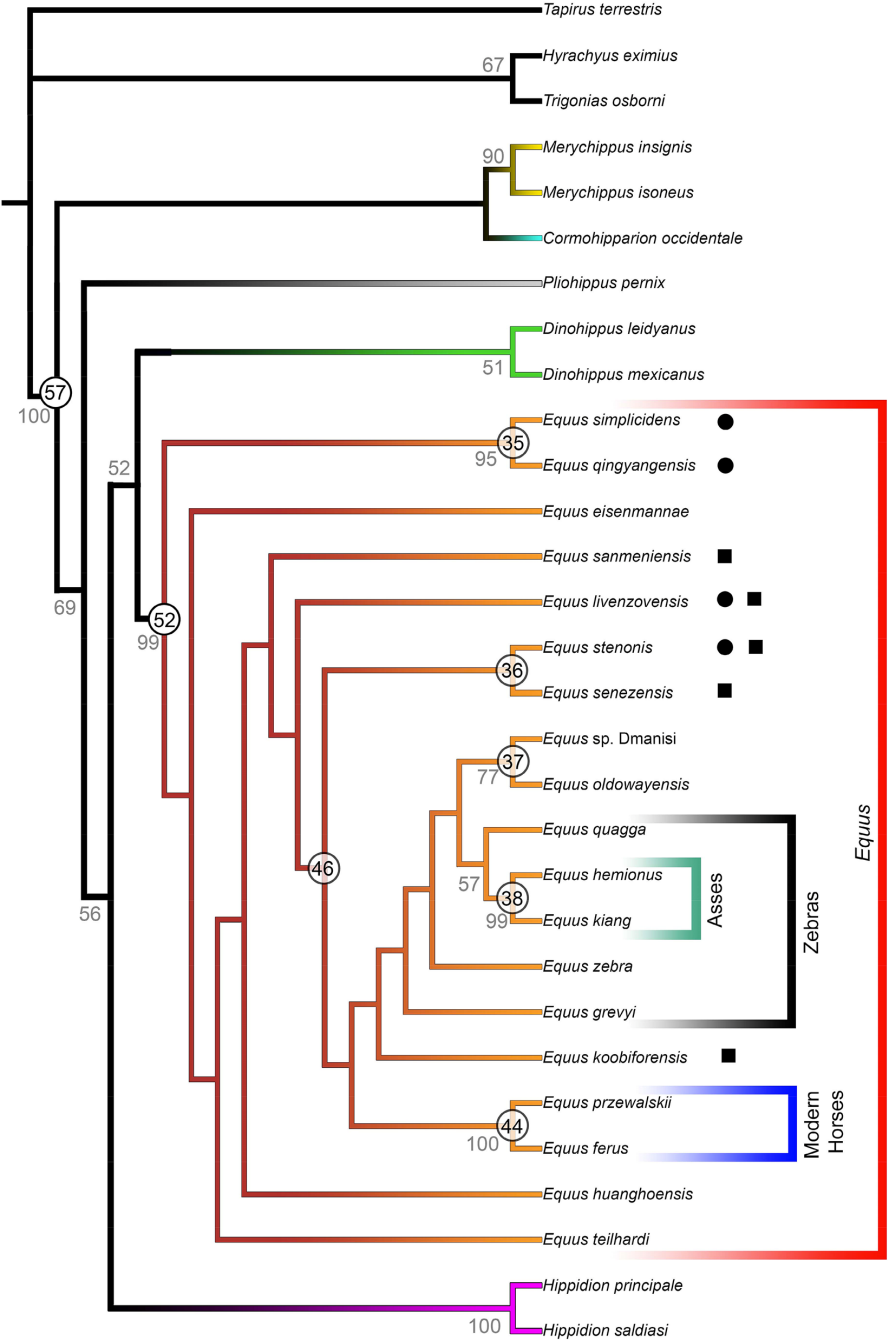
Most parsimonious tree of the cladistic analysis (30 taxa; 129 characters; Length = 398; CI = 0.472; RI = 0.705; HI = 0.528) recovered by PAUP 4.0β10^47^ and edited by Adobe Photoshop CC2017. Grey numbers report the Bootstrap values, whereas numbers included in circles represent the nodes discussed in the text. Terminal color branches indicate the following different genera identified by the cladistic analysis: yellow, *Merychippus*; Cyan, *Cormohipparion*; grey, *Pliohippus*; purple, *Hippidion*; green, *Dinohippus*; dark red to orange, *Equus*. On the right, red color box indicates the genus *Equus*, black box Zebras, dark green box Asses and blue box Modern Horses. Black circles indicate the previous genus attribution to *Plesippus*, whereas black squares *Allohippus*. The detailed analyses are reported in Appendix 1.

The cladistic analysis includes 30 Operative Taxonomic Units (OTU, with 4 outgroups and 26 ingroups) and 129 characters and it has produced one most parsimonious tree (Fig. 2) (Tree Length = 398 steps, Consistency Index = 0.472, Retention Index = 0.705; Homoplasy Index = 0.528). The characters have been coded by direct observations on fossil collections combined with other published fossil specimens (see Methods below). The present phylogenetic tree clusters the family Equidae by node 57 with 13 unambiguous synapomorphies (Appendix 1) and, furthermore, the Miocene tridactyl genera *Merychippus* and *Cormohipparion* are segregated from the monodactyl genus *Pliohippus* by 6 unambiguous synapomorphies (Appendix 1). The species referred to the genera *Merychippus*, *Hippidion* and *Dinohippus* are clustered together as dichotomies, with *Cormohipparion* being sister to *Merychippus*.

The phylogenetic tree reveals outcomes for the Plio-Pleistocene species from North America and the Old World. Genus *Equus* is modeled as a single clade with node 52 being supported by 18 unambiguous synapomorphies, and 13 of these have a CI ≥ 0.500. The complete list is reported in Table 1.

**Table 1:** List of unambiguous synapomorphies of the *Equus* clade (node 52 in Fig. 2).

Character description	Character number	Character state	Character state description	Consistency Index
Length of the skull	1	3	Between 500 and 550 mm	0.385
Lateral outline of skull	2	0	Linear	0.500
Buccinator fossa	16	1	Absent or poorly developed	1.0
Orbits position related to the upper third molar	27	3	Well behind the upper third molar	0.375
Lingual margin of the protocone	55	2	Shallow depression on its medial aspect	0.500
Protocone shape of the upper second premolar	60	1	Squared	0.500
Hypocone shape of the upper third and fourth premolar	61	1	Squared	0.167
Pli caballin shape of the upper third and fourth premolar	62	2	Present and elongated	1.0
Protocone shape of the upper third and fourth premolar	63	1	Squared	0.667
Hypocone shape of the upper first and second molar	64	1	Squared	0.143
Upper premolar cheek teeth length	71	2	Between 100 and 110 mm	0.375
Morphology of the metaconid-metastylid complex	91	2	V-shaped	1.0
Morphology of the lingual side of the metastylid	92	2	Squared	1.0
Functional morphology of foot	114	2	Hoof without soft pad, 3rd phalanx is strong and broad	0.500
Elongation of the lateral second metacarpal	116	3	Reduced up to the diaphysis and the proximal epiphysis of the third metacarpal	0.750
Elongation of the lateral fourth metacarpal	118	3	Reduced up to the diaphysis and the proximal epiphysis and the third metacarpal	0.750
Elongation of the lateral second metatarsal	122	3	Reduced up to the diaphysis and the proximal epiphysis of the third metatarsal	1.0
Elongation of the lateral fourth metatarsal	124	3	Reduced up to the diaphysis and the proximal epiphysis of the third metatarsal	1.0

The bootstrap tree supports the *Equus* clade with 99/100 replications (Appendix 1, bootstrap tree and UPGMA tree). The species previously included within the genera *Plesippus* and *Allohippus* are not clustered from the *Equus* clade, identified by the node 52 (Fig. 2). This evidence is strongly supported by the bootstrap resampling analyses and tree, where the species included in the clade *Equus* are grouped as polytomies, except for 4 small clades (Appendix 1, bootstrap tree). These small clades cluster the North American species *E. simplicidens* with the Chinese *E. qingyangensis* (node 35; bootstrap values 95/100), the *Equus* sp. from Dmanisi (Georgia, Caucasus) with the fossil African *E. oldowayensis* (node 37; bootstrap values 77/100), the extant *E. hemionus* and *E. kiang* (node 44; bootstrap values 99/100) and *E. przewalskii* and *E. ferus* (node 44; bootstrap values 100/100). These subclades do not represent other genera in the *Equus* clade (node 52), but may indicate no relevant morphological difference between these species being scored with similar character states (Table S1). An analogous result was already highlighted in recent research applying Geometrics Morphometrics on cranial elements in extant species^31^.

Notably, *E. simplicidens* and *E. qingyangensis* differ only by a single character, the shape of palatine process (slender in the former and flat in the Chinese species), raising a question about the validity of *E. qingyangensis*. Another small clade including *E. stenonis* and *E. senezensis* in the parsimonious tree (node 36, Fig. 2) is not supported by the bootstrap resampling (Appendix 1).

**Figure 3:**
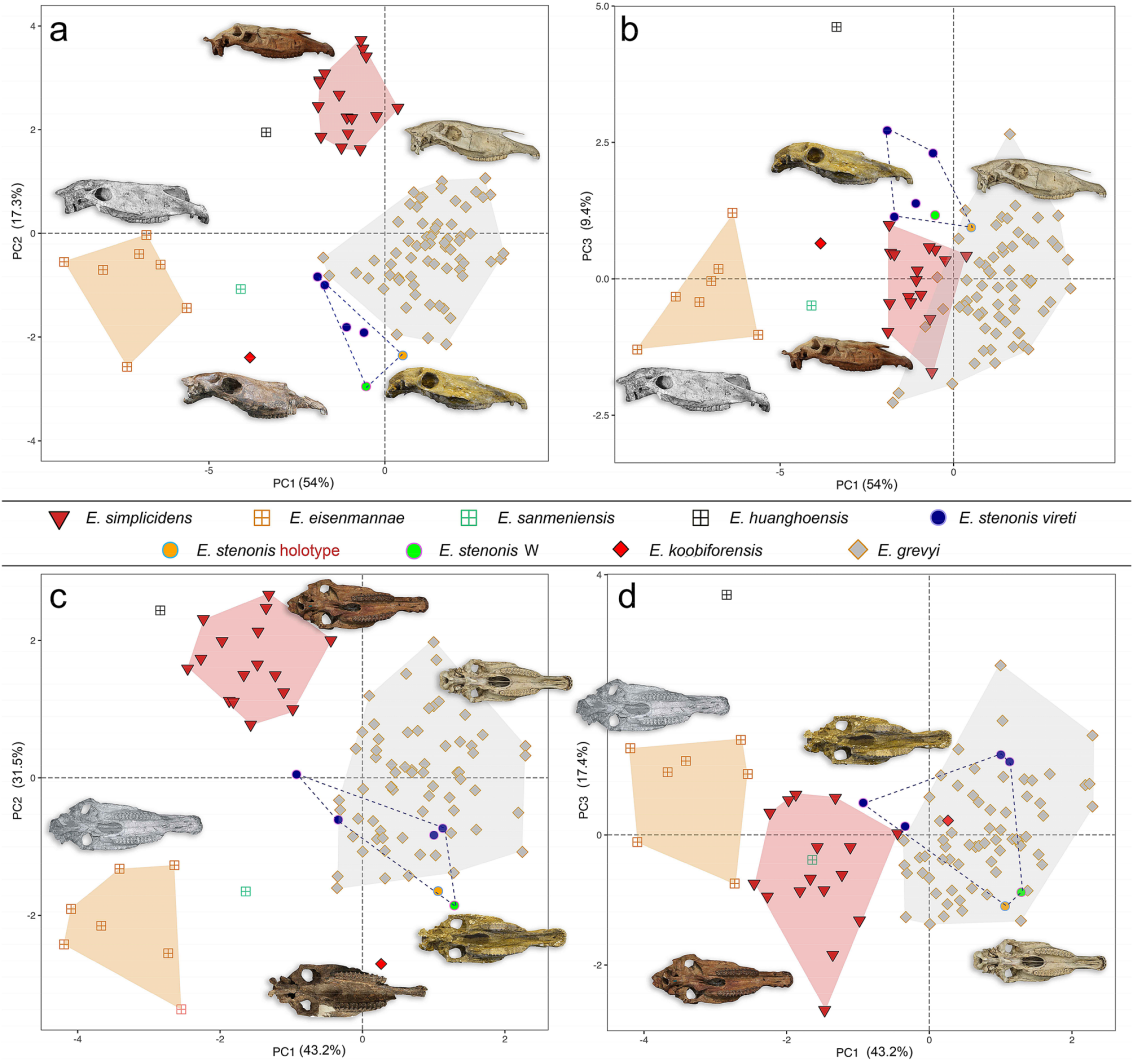
Results on PCA based on the selected skull measurements. Plots primarily obtained by R Studio Software v.1.4.103 2020^50^ packages *ggplot2()* v.3.3.3^51^ and *prcomp()* v.3.6.2^52^. (**a**, **b**) show the PC1 versus PC2-PC3 outcomes on the skull basal and lateral measurements, whereas (**c**, **d**) show the PC1 versus PC2-PC3 results for the skull basal measurements. The analyses have been developed by the Software R using the packages *prcomp()* and *ggplot2().* The original database is given in Table S3, whereas the loadings plots are shown in Figure S3.

Furthermore, the UPGMA tree based on the qualitative and quantitative characters described in the Table S1 may share new insights on the possible species relationships included in the *Equus* clade. The UPGMA well clusters the *Equus* clade from the other fossil genera of North and South America since the node 59 and, remarkably, separate the caballine horses (including *E. przewalskii*–*E. ferus*) from all other stenonine species (Appendix, UPGMA, node 58). This last cluster includes the entire fossil species from the New and the Old World and the extant zebras and asses. Noteworthy, the morphometric analyses based on the skull morphology show a similar result. The small clades within *Equus* evidenced by the most parsimonious tree and the bootstrap resampling are also present in the UPGMA tree (*E. simplicidens*–*E. qingyangensis*; *Equus* sp. Dmanisi–*E. oldowayensis* (Olorgesaile); *E. hemionus*–*E. kiang*; *E. przewalskii*–*E. ferus*) thus supporting their morphological similarities. The Early Pleistocene Chinese species *E. eisenmannae* is grouped with the *E. simplicidens*–*E. qingyangensis* clade, whereas, as reported in Fig. 2, *E. livenzovensis* and *E. stenonis* are the closest relatives of *E. koobiforensis* (Appendix 1, UPGMA tree). These relationships are reflected in the morphometric results on skulls, wherein *E. koobiforensis* is found to be closely related to *E. sanmeniensis* and *E. stenonis* (Fig. 3, S. text and Fig. S2). *Equus* sp. from Dmanisi (*Equus* aff. *E. altidens*^35^) and *E. oldowayensis* are still closely related^11^.

In our parsimonious tree (Fig. 2), *E. quagga* is regarded as a sister species of the *E. hemionus*–*E. kiang* clade. This result seems to be in contrast with the most recent molecular phylogenies^32–34^, wherein plain zebras and wild Asian asses are well clustered as distinct clades, indicting a remarkable difference in their genome sequences. Our outcomes may indicate a close morphological similarity in cranial and postcranial elements of the skeleton, which can be the results of multiple evolutionary or ecological factors and which will deserve future considerations and investigations.

Eventually, our analyses allow to support that the Plio-Pleistocene North American, Eurasian and African species can be all grouped within the genus *Equus*, and no distinction can be recognized for the genera *Plesippus* and *Allohippus*.

### Morphometric analysis

PCA results are based on total (basal and lateral) selected measurements (Fig. 3a,b), and basal measurements of the skull (Fig. 3c,d; see S. text for skull measurement references). Considering basal and lateral skull measurements (Fig. 3a), PC1 and PC2 accounts for most of the variance with 71.3% (PC1 = 54.0% and PC2 = 17.3%). The loadings’ distribution is shown in Figure S3 and reported in Table S2. PC1 separates species by maximum length (M6 and M23) from negative to positive values (more to less elongated), whereas PC2 mostly clusters species by M3 and M31 in positive values, and M4 and M5 negative values. The opposite development of the vomerine length (M3) and post vomerine length (M4) shown in the PCA is given also in the Log10 Ratio diagrams outcomes (Figure S2): even if *E. simplicidens*, *E. stenonis* and *E. grevyi* show the same skull length, *E. simplicidens* is separated from *E. stenonis* and *E. grevyi* by its greatly elongated vomerine length (M3) and a reduced post vomerine length (M4), whereas *E. stenonis* and *E. grevyi* show a longer development of the post vomerine length (M4) and a reduced vomerine length (M3). M30 and M31 cluster *E. simplicidens* and *E. stenonis*, with *E. grevyi* which occupies an intermediate morphospace between these species. This evidence is supported also by the Log10 Ratio diagrams (Figure S2), with *E. grevyi* showing a skull basal morphology similar to *E. stenonis*, and the lateral, naso-incisival notch, and cheek tooth length being similar to *E. simplicidens*. The Old World *Equus* species exhibit a longer naso-incisival notch dimension when compared to *E. grevyi*. *Equus koobiforensis* plots between *E. stenonis*, *E. sanmeniensis* and *E. eisenmannae*, even if the basal morphology of the skull appears to be more related to *E. stenonis* and *E. sanmeniensis* rather than *E. eisenmannae*. Nevertheless, its position in this diagram is influenced by the total maximum length (M6) and the upper cheek tooth row length (M7, M8, M9) which are slightly longer than *E. stenonis* and *E. grevyi* (see the original raw data in Table S3). The Chinese species *E. eisenmannae* and *E. huanghoensis* appear to be more closely related to *E. simplicidens* than *E. stenonis*. PC1 and PC3 account the 63.4% of the total variance (PC1 = 54.0% and PC3 = 9.4, Fig. 3b; the loadings’ distribution is shown in Figure S3b and reported in Table S2). PC1 separates species by M4 in positive values and maximum lengths (M6 and M23) in negative values (more to less elongated), whereas PC2 mostly clusters species by M5 and M31 with negative values (more to less elongated). In this diagram, *E. simplicidens*, *E. stenonis* and *E. grevyi* are closely clustered, overlapping some portions of their morphospaces. *Equus koobiforensis* plots between *E. stenonis*, *E. sanmeniensis* and *E. eisenmannae*, whereas *E. huanghoensis* is well separated from the entire sample by its reduced M5 and its elongated M2.

The PCA results on the basal skull measurements (Fig. 3c,d) do not include maximum skull length (M6). We have excluded this measurement in order to investigate the evolution of the basal skull morphology. PC1 and PC2 account for most of the variance with 74.7% (PC1 = 43.2% and PC2 = 31.5%, Fig. 3c; the loadings distribution is shown in Figure S3c and it is reported in Table S2). PC1 separates species by M1 (ventral length of the muzzle) and M2 (palatal length), from negative to positive values (more to less elongated), whereas PC2 mostly clusters species by M3 in positive and M4–M5 in negative values. These results are congruent with the previous clustering pattern (Fig. 3a). *Equus simplicidens* and *E. huanghoensis* are clustered by their longer M3 length, whereas *E. grevyi* and *E. stenonis* show higher values for M4. Nevertheless, *E. stenonis* overlaps with *E. grevyi*’s morphospace, providing additional support of the evidence shown in the Log10 Ratio diagram (Figure S2b). *Equus koobiforensis* is placed closer to *E. stenonis* and extant *E. grevyi*, whereas the Chinese species *E. eisenmannae* is the largest horse of the entire sample and *E. sanmeniensis* is placed between *E. eisenmannae* and *E. stenonis*. PC1 and PC3 account the 60.6% of the total variance (PC1 = 43.2% and PC3 = 17.4%, Fig. 3d; the loadings’ distribution is shown in Figure S3d and reported in Table S2). PC1 separates species by M4 with positive values and M1 and M3 with negative values (more to less elongated), whereas PC3 clusters species by M2 with positive values and M5 with negative values (more or less elongated). *Equus stenonis* and *E. grevyi* overlap extensively in their morphospaces which likewise include *E. koobiforensis*. Also, the *E. simplicidens* sample is placed close to *E. stenonis*, even if separated by the latter by its longer vomerine length (M3), and it includes *E. sanmeniensis* in its morphospace. *Equus eisenmannae* is more closely related to *E. simplicidens* than *E. stenonis*, supporting observations of the Log10 ratios diagrams (Figure S2a). *Equus huanghoensis* still remains separated from the entire sample by its reduced M5 and its elongated M2.

## Discussion

### Origin and early evolution of the *Equus grevyi* clade

**Figure 4:**
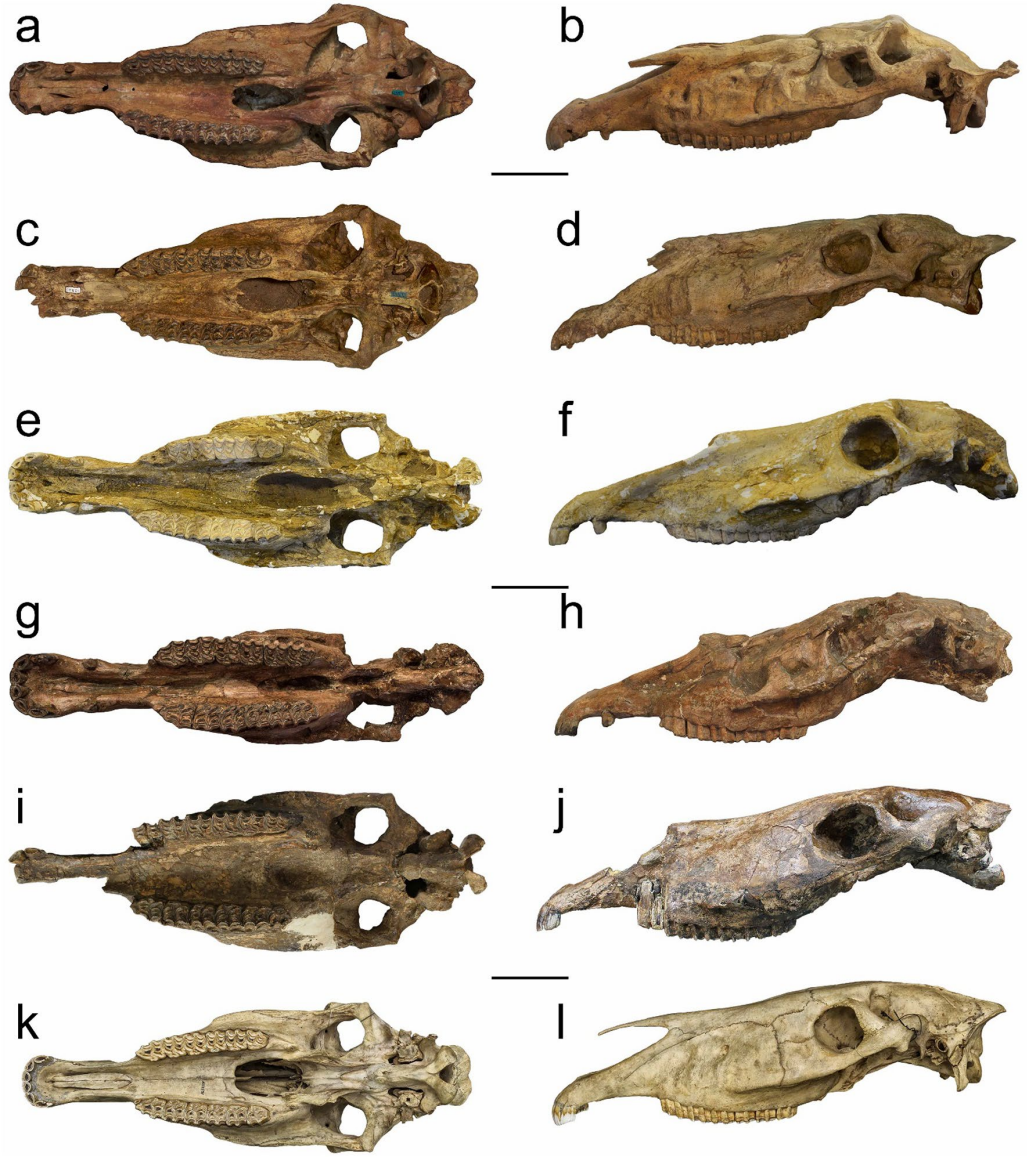
Comparisons of skulls, ventral and lateral views: *E. simplicidens* (USNM12573 and USNM12542, (**a**–**d**), *E. stenonis* (NHML20.163360 and IGF560 (**e**–**h**), *E. koobiforensis* (KNM-ER1284, i-j) and *E. grevyi* (USNM163228 k-l). Scale bar 10 cm.

Our phylogenetic and morphometric analyses, within the systematic position of *Equus stenonis*, provide novel insights into the phylogenetic relationships of the Old World *Equus* and the origin of the zebra-ass clade based on paleontological evidence^2–4,7,11,12,15,18–22,36,37^. As reported in the outcomes shown by the morphometric analyses, the Early Pleistocene Chinese species *E. eisenmannae, E. qingyangensis* and *E. huanghoensis* have more primitive skull traits than *E. stenonis* that compare best with North American *E. simplicidens*, whereas the African *E. koobiforensis* and the extant *E. grevyi* are more closely related to *E. stenonis* and *E. sanmeniensis*. Nevertheless, *Equus grevyi* has a reduced muzzle when compared to the fossil species (Fig. 4l). The *E. simplicidens* skull is characterized as having a longer vomer length and a reduced post vomerine length (Fig. 4a,c), whereas *E. stenonis*, *E. koobiforensis* and *E. grevyi* have a reduced vomer length and a longer post vomerine length (Figs. 4e,g,i,k). In lateral view, *E. simplicidens* (Fig. 4b,d) has an elongated skull with a linear dorsal outline and a deep incision of the narial notch, whereas the skulls of *E. stenonis* (Fig. 4f,h) and *E. koobiforensis* (Fig. 4j) have a concave dorsal skull outline, akin to *E. grevyi* (Fig. 4l).

**Figure 5:**
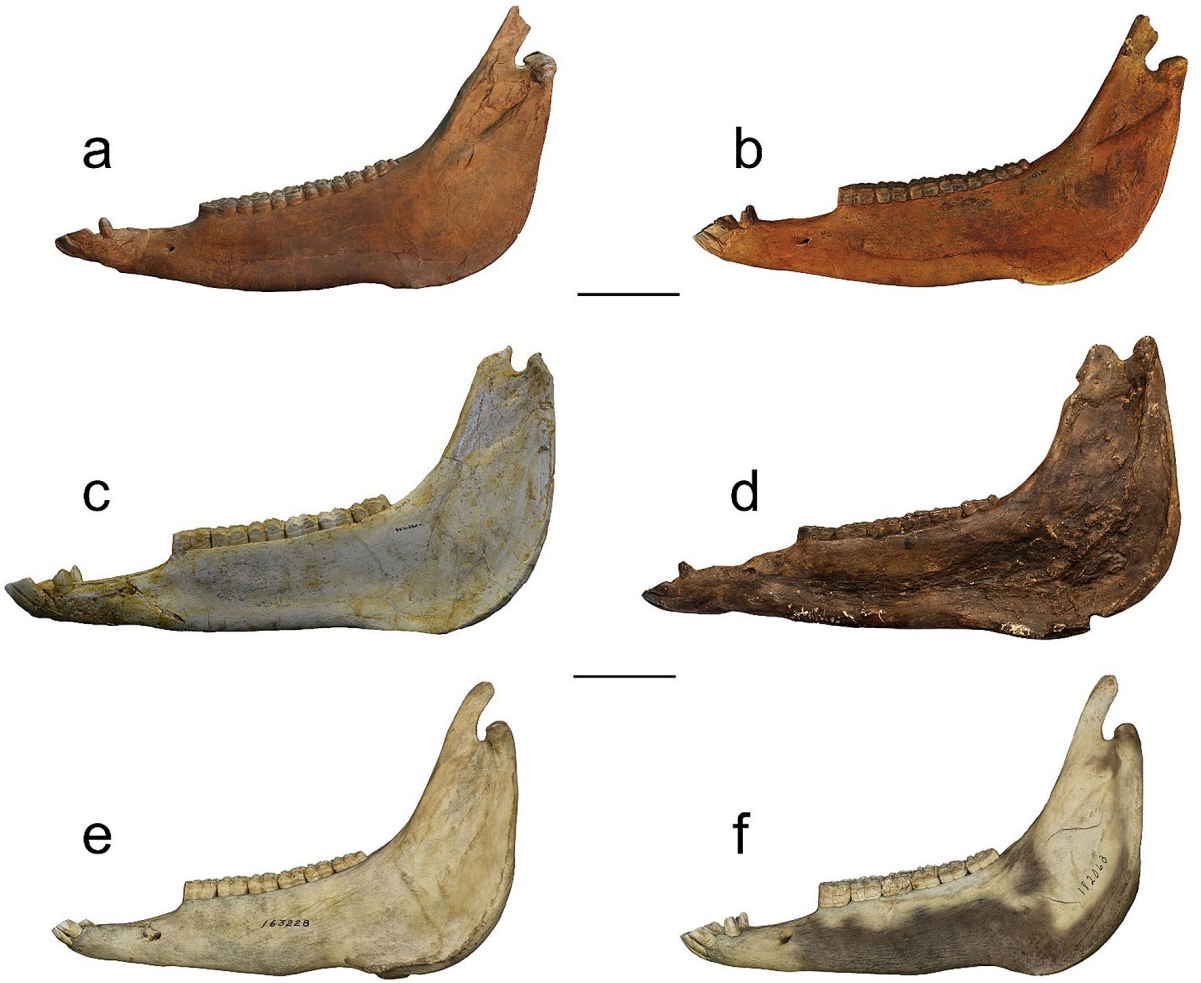
Mandible morphological comparison of *E. simplicidens* (USNM12573 and USNM12522, (**a**,**b**), *E. stenonis* (NHML20.163361 and IGF560, (**c**,**d**), and *E. grevyi* (USNM163228 and USNM182063 (**e**,**f**). Scale bar 10 cm.

Furthermore, this skull development could be related to the mandibular profile. *Equus simplicidens* has the mandibular ramus angled posteriorly, whereas that of *E. stenonis* is vertically oriented (Fig. 5a–d). There is no mandible associated with *E. koobiforensis*. *Equus grevyi* has a mandible shaped more like *E. stenonis*, with a steep vertical ramus and a large and round posterior angle of the mandible (Fig. 5e–f).

The preorbital fossa (POF) underwent progressive reduction in *Equus* species related to the increase in cheek tooth crown height^7,11–14^. Figure S4 summarizes POF evolution in *E. simplicidens, E. eisenmannae, E. stenonis, E. koobiforensis* and *E. grevyi*, in the lateral morphology of the skull by its perpendicular maximum height (M35), by distance between POF and the facial maxillary crest (M36) and by its height of the back of the POF above the alveolar border (M38). The POF is larger in *E. simplicidens, E. eisenmannae* and *E. stenonis* (M35) but it is placed higher on the maxilla (M36 and M38) in *E. stenonis, E. koobiforensis* and *E. grevyi. Equus stenonis* exhibits both plesiomorphic characters as does *E. simplicidens* and *E. eisenmannae* (M35)*,* and more derived characters as seen in *E. koobiforensis* and *E. grevyi* (M36, M38) (S. text and Figure S4).

Recent research^38,39^ cites *Equus’* distinction as having the greatest crown height of all Equidae. In turn, increased hypsodonty is hypothesized to be an adaptation to more arid environments in the Early Pleistocene (a generalized Neogene trend in ungulates^40^). Horses became more adapted to grazing during the Pleistocene with a higher degree of hypsodonty and, as a consequence, increased hypsodonty also affected both the development of the lateral shape of the skull and the expression of the POF. Such evidence is provided by the evolution of the preorbital fossae (Fig. 3 and Figure S4), which is strongly reduced in *E. grevyi* and *E. koobiforensis* when compared to *E. simplicidens*.

Furthermore, there are important and consistent traits of the cheek tooth dentitions in *Equus.* The lingual margin of the protocone has a shallow depression on its medial aspect in *E. simplicidens* and *E. stenonis*, and it is more evident in *E. koobiforensis* and *E. grevyi* (S. text and Figure S5a–d; Ch. 55 in Table S1). In the lower cheek tooth row, the typical V-shaped linguaflexid and stenonine metaconid-metastylid morphology is precociously present in *E. simplicidens* (S. text and Figure S5f–h; Ch. 91 in Table S1). However, the remarkable squared morphology of the lingual margin of the metastylid is found in *E. stenonis*, *E. koobiforensis* and extant *E. grevyi*, and it is present, even if less clear, in *E. simplicidens* (S. text and Figure S5f–h; Ch. 92 in Table S1).

### Evolutionary remarks

**Figure 6:**
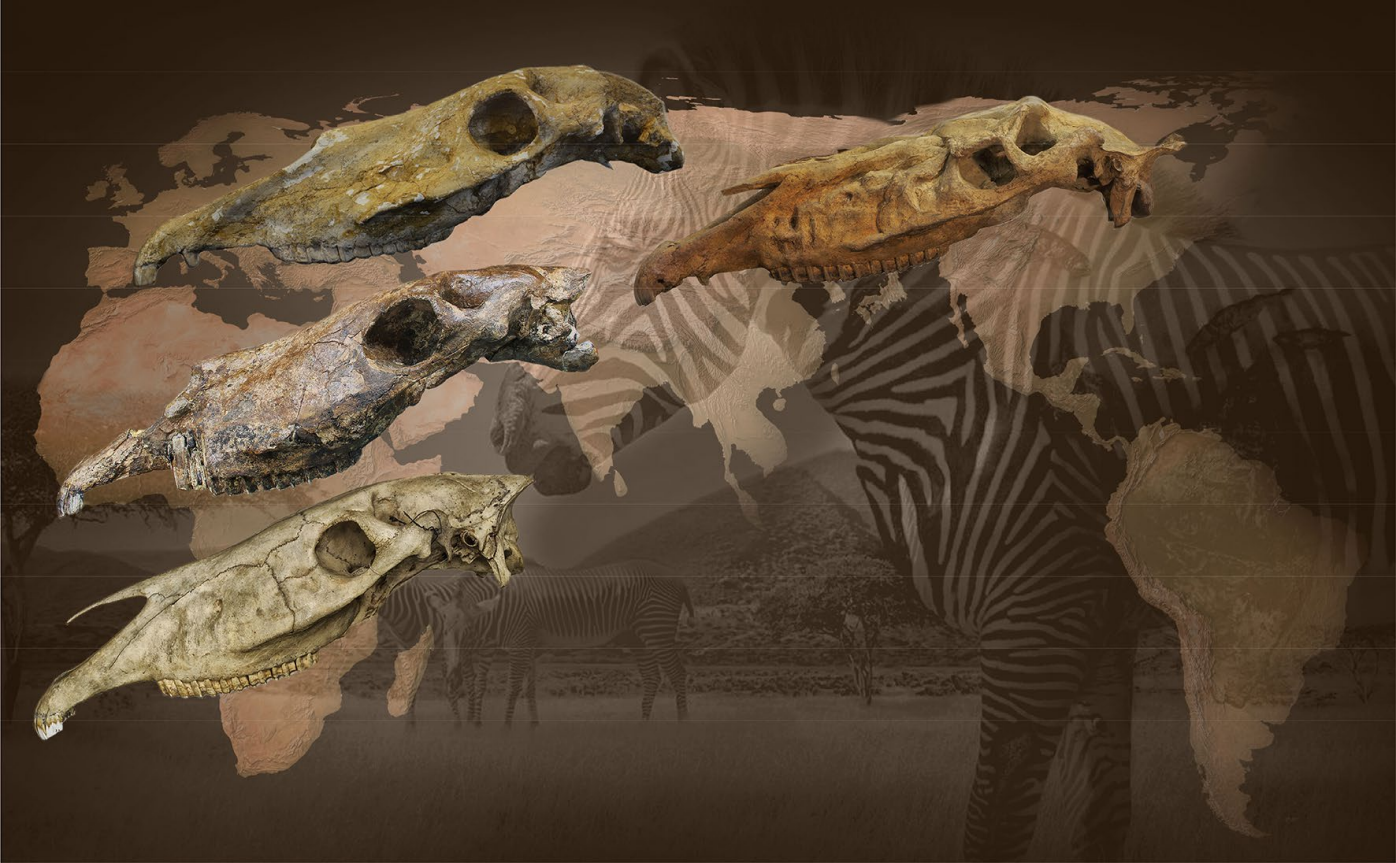
Evolutionary framework of the extant zebras *Equus grevyi* since the oldest common ancestor *Equus simplicidens*, and through the European *Equus stenonis* and the African *Equus koobiforensis*. The present figure aims to represent the dispersal of the genus *Equus* in the Old World by *E. simplicidens* at the beginning of the Pleistocene, and the origin of the extant zebras *E. grevyi* through the *E. stenonis* and *E. koobiforensis* lineage. Artwork O. Cirilli (map edited from row data of QGIS v.3.18.1 (https://www.qgis.org/it/site/), zebras in background edited from Nina Marie Photography (https://ninamarievisuals.com/)).

The various analyses provided herein have shown that the evolution of the head morphology occurred in the evolutionary steps *E. simplicidens*–*E. stenonis*–*E. koobiforensis*–*E. grevyi* + zebra-ass clade (Fig. 6) including: (i) the reduction of the vomerine length; (ii) elongation of the post vomerine length; (iii) reduction of the length of the naso-incisival notch; (iv) progressive reduction of the POF, from a large and more developed structure to a reduced and shallow morphology, which is still present in the extant zebras; (v) progressively more vertically oriented mandibular ramus; (vi) a more derived morphology of the lingual margin of the protocone; (vii) the advanced squared shape of the metastylid and persistent V- shaped linguaflexid .

Following Azzaroli and Voorhies^5^, we find that North American Pliocene *Equus simplicidens* represents the likely ancestral stock for the origin of Eurasian stenonine horses and ultimately African *E. grevyi,* and the zebra-ass clade^1–6,9,11,18,41^. Our phylogenetic results support *Equus* as being a single clade, with *Dinohippus* as the sister taxon. Our results do not support *Plesippus* and *Allohippus* at either the generic or subgeneric ranks. Our phylogenetic outcomes support the most recent genomic outputs^9,33^, which have found evolutionary rates for the *Equus* most common recent ancestor living 3.6–5.8 Ma. Ancient DNA analyses have shown slower mutation rates in horses than humans^9^ implying a minimal date of 4.07 Ma for *Equus’* most common recent ancestor, proposing an age of 4.0–4.5 Ma for the origin of *Equus.* The concurrent evidence of our phylogenetic and the genomic results^9^ can be correlated with the most recent paleontological findings in Central America^21,36^, which have proved the occurrence of the primitive *Equus* morphologies^36^ at the Hemphillian–Blancan boundary at ca. 4 Ma^19,21,36^ correlative with the onset of Pliocene global warming^11,19,36,42^.

## Methods

### Phylogenetic analysis

Our cladistic analysis uses 30 Operational Taxonomic Units (OTUs) and 129 characters (72 cranial, 40 mandibular and 17 on autopodia), including 26 equid taxa and 4 outgroups, *Tapirus terrestris*, *Hyrachyus eximius*, *Trigonias osborni* and *Merychippus insignis* (Appendix 1 and Table S1). The complete sample of the specimens coded in the cladistic matrix is reported in S. text. The matrix used in this cladistic analysis includes 68 novel characters, combined with the most recent matrices for equids and perissodactyl cladistic analyses^12,20,43–46^ (Table S1). The characters have been coded by direct observations. 24 characters are ordered (2, 5, 9, 10, 11, 12, 13, 14, 16, 22, 23, 42, 43, 52, 78, 91, 92, 113, 114, 116, 118, 121, 123, 129) and 105 characters unordered. All characters were equally weighted. The phylogenetic analysis was undertaken using PAUP 4.0β10^47^, under parsimony using Heuristic Search with the TBR (tree bisection reconnection) branch-swapping algorithm, 1000 bootstrap replications with additional random sequence, gaps treated as missing.

### Morphometric analysis

We have undertaken statistical analyses (Log10 ratio diagrams, PCA, and boxplots) on selected skull, mandible and dental morphologies and measurements (S. text) to evaluate our cladistic analysis of Plio-Pleistocene Holarctic and African *Equus* evolution. We use international equid measurement standards^48,49^. Plots were generated primarily using the R Studio Software v.1.4.103 2020^50^ packages *ggplot2()* v.3.3.3 and *prcomp()* v.3.6.2, using the function (scale = T). Following previous analytical studies^7^, we have selected skull length measurements, avoiding medio-lateral deformation which would adversely influence our results. The complete database used in the Morphometric analyses is reported in Table S3, including personal and other published available data^53^–^56^.

## Supplementary Information

Supplementary Information 1.

Supplementary Information 2.

Supplementary Information 3.

Supplementary Information 4.

Supplementary Information 5.
