# Supplementary material for: Evolution of Old World Equus and origin of the zebra-ass clade
Source: Sci Rep. 2021 May 12;11:10156. doi: 10.1038/s41598-021-89440-9 (PMC8114910; doi:10.1038/s41598-021-89440-9)
Supplement: Supplementary file 1 — Supplementary Information 1. [file 41598_2021_89440_MOESM1_ESM.doc]

**Analysis *Equus***

Heuristic search, stepwise additional option with random sequence = 1000.

Heuristic search settings:

Optimality criterion = parsimony

Character-status summary:

Of 129 total characters:

24 characters are of type 'ord' (Wagner)

105 characters are of type 'unord'

All characters have equal weight

23 characters are constant

5 variable characters are parsimony-uninformative

Number of parsimony-informative characters = 101

Gaps are treated as "missing"

Starting tree(s) obtained via stepwise addition

Addition sequence: random

Number of replicates = 1000

Starting seed = 418195301

Number of trees held at each step during stepwise addition = 1

Branch-swapping algorithm: tree-bisection-reconnection (TBR)

Steepest descent option not in effect

Initial 'MaxTrees' setting = 100

Branches collapsed (creating polytomies) if maximum branch length is zero

'MulTrees' option in effect

Topological constraints not enforced

Trees are unrooted

Heuristic search completed

Total number of rearrangements tried = 34043232

Score of best tree(s) found = 398

Number of trees retained = 1

Tree # 1

Length 398

CI 0.472

RI 0.705

RC 0.333

HI 0.528

G-fit -68.500

*/------------------------------------------------------------- Tapirus terrestris*

*|*

*| /--- Hyrachyus eximius*

*+--------------------------------------------------------31*

*| \--- Trigonias osborni*

*|*

*| /--- Merychippus insignis*

*| /-32*

*| | \--- Merychippus isoneus*

*58 /--------------------------------------------------33*

*| | \------ Cormohipparion occidentale*

*| |*

*| | /------------------------------------------------------- Pliohippus pernix*

*| | |*

*| | | /--- Dinohippus leidyanus*

*| | | /-------------------------------------------34*

*| | | | \--- Dinohippus mexicanus*

*| | | |*

*\-57 | | /--- Equus simplicidens*

*| | | /----------------------------------------35*

*| | | | \--- E. qingyangensis*

*| | /-53 |*

*| | | | | /------------------------------------------ E. eisenmannae*

*| | | | | |*

*| | | | | | /-------------------------------- E. sanmeniensis*

*| | | | | | |*

*\-56 | | | | | /----------------------------- E. livenzovensis*

*| | \-52 | | |*

*| | | | /-48 | /--- E. stenonis*

*| | | | | | | /---------------------36*

*| | | | | | | | \--- E. senezensis*

*| | | | | | | |*

*| | | | | \-47 | /--- E. sp. Dmanisi*

*| | | | | | | /-----37*

*| | | | | | | | \--- E. oldowayensis*

*| | \-51 | | | |*

*| | | | | | /-40 /------ E. quagga*

*| | | | | | | | |*

*| | | | \-46 | \--39 /--- E. hemionus*

*\--55 | | | /-41 \-38*

*| | /--49 | | | \--- E. kiang*

*| | | | | | |*

*| | | | | /-42 \------------- E. zebra*

*| | | | | | |*

*| | | | | /-43 \---------------- E. grevyi*

*| | | | | | |*

*| | | | | | \------------------- E. koobiforensis*

*| \-50 | \--45*

*| | | | /--- E. przewalskii*

*| | | \-----------------44*

*| | | \--- E. ferus*

*| | |*

*| | \----------------------------------- E. huanghoensis*

*| |*

*| \--------------------------------------- E. teilhardi*

*|*

*| /--- Hippidion principale*

*\----------------------------------------------54*

*\--- Hippidion saldiasi*

Apomorphy lists:

green: synapomorphies of the family Equidae

grey: synapomorphies of the Monodactyl clade Equinae

yellow: synapomorphies of the *Equus* clade

Branch Character Steps CI Change

----------------------------------------------------------------------

node_58 --> Tapirus terrestr 4 1 0.333 0 --> 1

7 1 0.417 0 --> 6

41 1 0.400 1 ==> 0

56 1 0.500 0 --> 1

75 1 0.500 0 --> 1

node_58 --> node_31 23 1 0.500 1 ==> 2

35 1 1.000 0 ==> 1

36 1 0.500 1 ==> 0

44 1 1.000 0 ==> 1

76 1 1.000 0 ==> 1

node_31 --> Hyrachyus eximiu 17 1 0.500 0 --> 1

78 1 0.333 0 ==> 1

node_31 --> Trigonias osborn 1 1 0.385 0 ==> 3

3 1 1.000 0 ==> 1

4 1 0.333 0 --> 1

6 1 0.500 0 ==> 1

7 1 0.417 0 --> 1

32 1 0.500 0 --> 1

34 1 1.000 0 ==> 1

55 1 0.500 2 ==> 3

73 1 0.667 0 --> 2

75 1 0.500 0 --> 1

89 1 1.000 0 ==> 1

113 1 0.667 0 --> 1

node_58 --> node_57 5 1 0.667 0 --> 1

9 2 0.667 2 ==> 0

17 1 0.500 0 --> 1

18 1 1.000 0 ==> 2

20 1 1.000 0 ==> 2

27 1 0.375 0 ==> 1

29 1 1.000 0 ==> 1

31 1 1.000 0 ==> 1

32 1 0.500 0 --> 1

39 1 0.231 1 --> 2

43 1 0.333 1 ==> 0

54 1 0.500 0 --> 1

55 1 0.500 2 ==> 1

56 1 0.500 0 --> 2

57 1 0.500 1 --> 0

62 1 1.000 0 ==> 1

65 1 0.500 0 ==> 1

68 1 0.250 1 --> 0

69 1 1.000 0 ==> 1

73 1 0.667 0 --> 2

77 1 1.000 0 ==> 1

81 1 1.000 0 ==> 1

83 1 0.500 0 --> 1

102 1 0.333 1 --> 0

107 1 0.167 1 --> 0

113 1 0.667 0 --> 1

114 1 0.500 0 --> 1

node_57 --> node_33 7 1 0.417 0 --> 1

36 1 0.500 1 ==> 2

41 1 0.400 1 ==> 2

51 1 0.667 0 --> 2

52 1 0.500 1 ==> 0

53 1 1.000 1 ==> 0

58 1 0.333 0 ==> 1

90 1 0.250 0 --> 1

116 1 0.750 1 ==> 0

118 1 0.750 1 ==> 0

122 1 1.000 1 ==> 0

124 1 1.000 1 ==> 0

126 1 0.429 0 --> 1

node_33 --> node_32 5 1 0.667 1 --> 0

8 1 0.500 1 --> 0

22 3 0.833 4 ==> 1

37 1 1.000 2 ==> 1

40 1 0.273 1 --> 2

54 1 0.500 1 --> 0

56 1 0.500 2 --> 1

57 1 0.500 0 --> 1

61 1 0.167 0 --> 1

64 1 0.143 0 --> 1

68 1 0.250 0 --> 1

73 1 0.667 2 ==> 1

84 1 1.000 3 ==> 1

95 1 1.000 0 --> 2

97 1 0.333 1 --> 0

110 1 0.333 1 --> 0

114 1 0.500 1 --> 0

120 1 0.429 1 ==> 2

126 1 0.429 1 --> 2

128 1 0.500 1 ==> 2

node_32 --> Merychippus insi 7 1 0.417 1 --> 0

23 1 0.500 1 ==> 0

39 1 0.231 2 --> 3

40 1 0.273 2 --> 3

node_32 --> Merychippus ison 22 1 0.833 1 ==> 0

79 1 0.500 0 ==> 1

node_33 --> Cormohipparion o 1 1 0.385 0 ==> 2

39 1 0.231 2 --> 1

55 1 0.500 1 ==> 0

59 1 0.400 0 ==> 1

67 1 0.250 1 ==> 0

70 1 1.000 0 ==> 3

71 1 0.375 0 --> 1

72 1 0.375 0 ==> 1

82 1 0.333 0 ==> 1

94 1 0.500 0 ==> 1

96 1 0.667 0 --> 2

99 1 0.500 0 ==> 1

100 1 0.500 1 ==> 0

101 1 0.667 0 ==> 1

102 1 0.333 0 --> 1

104 1 0.667 0 ==> 1

106 1 0.250 0 ==> 1

107 1 0.167 0 --> 1

109 1 0.500 0 ==> 1

111 1 0.333 0 ==> 1

119 1 0.300 3 ==> 2

125 1 0.300 3 ==> 2

127 1 0.750 2 ==> 1

node_57 --> node_56 2 1 0.500 0 ==> 1

22 1 0.833 4 --> 5

24 1 0.286 0 ==> 1

70 1 1.000 0 ==> 2

78 3 0.333 0 --> 3

79 1 0.500 0 ==> 1

91 1 1.000 0 --> 1

92 1 1.000 0 --> 1

105 1 0.167 1 --> 2

113 1 0.667 1 --> 2

116 1 0.750 1 --> 2

118 1 0.750 1 --> 2

127 1 0.750 2 --> 0

129 1 0.500 0 --> 1

node_56 --> Pliohippus perni 8 1 0.500 1 ==> 0

40 1 0.273 1 --> 2

59 1 0.400 0 ==> 2

node_56 --> node_55 6 1 0.500 0 ==> 1

7 1 0.417 0 --> 3

10 1 0.500 0 ==> 1

15 1 0.333 0 ==> 2

23 1 0.500 1 --> 2

25 1 0.333 0 ==> 1

27 1 0.375 1 ==> 2

39 1 0.231 2 --> 0

71 1 0.375 0 --> 1

72 1 0.375 0 ==> 2

82 1 0.333 0 --> 1

node_55 --> node_53 9 1 0.667 0 ==> 1

14 1 0.667 0 ==> 1

22 1 0.833 5 --> 4

66 1 0.500 0 ==> 2

68 1 0.250 0 --> 1

90 1 0.250 0 ==> 1

93 1 1.000 2 ==> 3

94 1 0.500 0 ==> 1

96 1 0.667 0 --> 2

98 1 0.333 0 --> 1

99 1 0.500 0 ==> 1

101 1 0.667 0 ==> 2

104 1 0.667 0 ==> 1

109 1 0.500 0 ==> 1

110 1 0.333 1 --> 2

119 1 0.300 3 --> 0

125 1 0.300 3 ==> 1

node_53 --> node_34 14 1 0.667 1 ==> 2

23 1 0.500 2 --> 1

24 1 0.286 1 --> 0

39 1 0.231 0 ==> 3

40 1 0.273 1 --> 3

72 1 0.375 2 ==> 1

82 1 0.333 1 --> 0

114 1 0.500 1 --> 0

116 1 0.750 2 --> 1

118 1 0.750 2 --> 1

119 1 0.300 0 --> 2

126 1 0.429 0 --> 2

127 1 0.750 0 --> 1

node_34 --> Dinohippus leidy 15 1 0.333 2 ==> 1

71 1 0.375 1 --> 0

100 1 0.500 1 ==> 0

105 1 0.167 2 --> 1

106 1 0.250 0 ==> 1

107 1 0.167 0 ==> 1

110 1 0.333 2 --> 1

120 1 0.429 1 ==> 2

125 1 0.300 1 ==> 2

127 1 0.750 1 --> 3

128 1 0.500 1 ==> 2

129 1 0.500 1 ==> 0

node_34 --> Dinohippus mexic 1 1 0.385 0 ==> 1

50 1 1.000 0 ==> 1

96 1 0.667 2 ==> 1

97 1 0.333 1 ==> 0

98 1 0.333 1 --> 0

node_53 --> node_52 1 1 0.385 0 ==> 3

2 1 0.500 1 ==> 0

16 1 1.000 0 ==> 1

27 1 0.375 2 ==> 3

55 1 0.500 1 ==> 2

60 1 0.500 0 ==> 1

61 1 0.167 0 ==> 1

62 1 1.000 1 ==> 2

63 1 0.667 0 ==> 1

64 1 0.143 0 ==> 1

71 1 0.375 1 --> 2

91 1 1.000 1 ==> 2

92 1 1.000 1 ==> 2

114 1 0.500 1 ==> 2

116 1 0.750 2 ==> 3

118 1 0.750 2 ==> 3

122 1 1.000 1 ==> 3

124 1 1.000 1 ==> 3

node_52 --> node_35 15 1 0.333 2 ==> 1

25 1 0.333 1 --> 0

59 1 0.400 0 ==> 1

119 1 0.300 0 --> 1

126 1 0.429 0 --> 1

node_35 --> Equus simplicide 39 1 0.231 0 --> 1

node_52 --> node_51 12 1 0.500 0 ==> 1

41 1 0.400 1 ==> 0

102 1 0.333 0 --> 1

105 1 0.167 2 --> 1

108 1 0.500 0 ==> 1

120 1 0.429 1 ==> 0

125 1 0.300 1 ==> 0

node_51 --> E. eisenmannae 1 1 0.385 3 ==> 5

10 1 0.500 1 ==> 2

40 1 0.273 1 --> 0

71 1 0.375 2 ==> 3

72 1 0.375 2 ==> 3

97 1 0.333 1 ==> 0

100 1 0.500 1 ==> 0

102 1 0.333 1 --> 2

103 1 0.500 0 ==> 1

107 1 0.167 0 ==> 1

110 1 0.333 2 --> 1

node_51 --> node_50 39 1 0.231 0 --> 1

66 1 0.500 2 ==> 1

67 1 0.250 1 ==> 0

78 1 0.333 3 --> 2

106 1 0.250 0 --> 1

128 1 0.500 1 ==> 0

node_50 --> node_49 1 1 0.385 3 ==> 4

61 1 0.167 1 ==> 0

node_49 --> node_48 2 1 0.500 0 ==> 1

42 1 0.500 0 ==> 1

43 1 0.333 0 ==> 1

node_48 --> E. sanmeniensis 10 1 0.500 1 ==> 2

36 1 0.500 1 ==> 0

72 1 0.375 2 ==> 3

105 1 0.167 1 ==> 2

node_48 --> node_47 64 1 0.143 1 ==> 0

68 1 0.250 1 ==> 0

107 1 0.167 0 ==> 1

111 1 0.333 0 ==> 1

node_47 --> E. livenzovensis 83 1 0.500 1 ==> 0

node_47 --> node_46 1 1 0.385 4 --> 3

66 1 0.500 1 ==> 2

119 1 0.300 0 ==> 1

125 1 0.300 0 ==> 1

node_46 --> node_36 7 1 0.417 3 ==> 4

node_36 --> E. senezensis 15 1 0.333 2 ==> 1

27 1 0.375 3 ==> 2

36 1 0.500 1 ==> 0

39 1 0.231 1 ==> 3

40 1 0.273 1 ==> 3

59 1 0.400 0 ==> 1

71 1 0.375 2 ==> 1

72 1 0.375 2 ==> 1

119 1 0.300 1 ==> 2

120 1 0.429 0 ==> 4

125 1 0.300 1 ==> 2

126 1 0.429 0 ==> 4

node_46 --> node_45 11 1 1.000 0 ==> 1

13 1 1.000 1 ==> 2

51 1 0.667 0 --> 1

65 1 0.500 1 --> 2

67 1 0.250 0 --> 1

94 1 0.500 1 ==> 2

99 1 0.500 1 ==> 2

109 1 0.500 1 ==> 2

node_45 --> node_43 39 1 0.231 1 ==> 0

55 1 0.500 2 ==> 3

64 1 0.143 0 ==> 1

105 1 0.167 1 ==> 2

node_43 --> node_42 40 1 0.273 1 ==> 0

41 1 0.400 0 ==> 1

120 1 0.429 0 ==> 1

126 1 0.429 0 ==> 1

node_42 --> node_41 7 1 0.417 3 ==> 2

27 1 0.375 3 --> 2

111 1 0.333 1 ==> 0

119 1 0.300 1 ==> 2

125 1 0.300 1 ==> 2

node_41 --> node_40 39 1 0.231 0 --> 1

58 1 0.333 0 --> 1

61 1 0.167 0 --> 1

66 1 0.500 2 ==> 1

102 1 0.333 1 --> 0

108 1 0.500 1 ==> 0

109 1 0.500 2 ==> 1

node_40 --> node_37 55 1 0.500 3 ==> 2

65 1 0.500 2 ==> 1

71 1 0.375 2 ==> 1

72 1 0.375 2 ==> 1

node_37 --> E. altidens Dman 15 1 0.333 2 ==> 1

64 1 0.143 1 ==> 0

node_37 --> E. oldowayensis 27 1 0.375 2 --> 3

40 1 0.273 0 ==> 1

52 1 0.500 1 ==> 2

node_40 --> node_39 1 1 0.385 3 ==> 1

7 1 0.417 2 --> 1

39 1 0.231 1 --> 2

40 1 0.273 0 ==> 2

78 2 0.333 2 ==> 4

94 1 0.500 2 ==> 1

98 1 0.333 1 ==> 0

99 1 0.500 2 ==> 1

106 1 0.250 1 ==> 0

107 1 0.167 1 ==> 0

node_39 --> E. quagga 24 1 0.286 1 ==> 0

41 1 0.400 1 ==> 0

102 1 0.333 0 --> 1

119 1 0.300 2 ==> 3

125 1 0.300 2 ==> 3

node_39 --> node_38 15 1 0.333 2 ==> 1

27 1 0.375 2 --> 3

39 1 0.231 2 --> 3

52 1 0.500 1 ==> 2

58 1 0.333 1 --> 0

61 1 0.167 1 --> 0

64 1 0.143 1 ==> 0

90 1 0.250 1 ==> 0

101 1 0.667 2 ==> 1

105 1 0.167 2 ==> 1

110 1 0.333 2 ==> 1

120 1 0.429 1 ==> 2

126 1 0.429 1 ==> 2

node_38 --> E. kiang 1 1 0.385 1 ==> 2

7 1 0.417 1 --> 2

node_41 --> E. zebra 1 1 0.385 3 ==> 2

24 1 0.286 1 ==> 0

63 1 0.667 1 ==> 2

node_42 --> E. grevyi 59 1 0.400 0 ==> 1

node_43 --> E. koobiforensis 1 1 0.385 3 --> 4

51 1 0.667 1 --> 0

61 1 0.167 0 ==> 1

65 1 0.500 2 --> 1

67 1 0.250 1 --> 0

node_45 --> node_44 1 1 0.385 3 ==> 2

7 1 0.417 3 ==> 2

12 1 0.500 1 ==> 0

14 1 0.667 1 ==> 0

24 1 0.286 1 ==> 0

27 1 0.375 3 ==> 2

43 1 0.333 1 ==> 0

52 1 0.500 1 ==> 2

60 1 0.500 1 ==> 0

63 1 0.667 1 ==> 2

78 2 0.333 2 ==> 4

90 1 0.250 1 ==> 0

91 1 1.000 2 ==> 3

103 1 0.500 0 ==> 1

104 1 0.667 1 ==> 2

110 1 0.333 2 ==> 0

node_44 --> E. przewalskii 39 1 0.231 1 ==> 3

40 1 0.273 1 ==> 3

119 1 0.300 1 ==> 3

125 1 0.300 1 ==> 3

node_49 --> E. huanghoensis 7 1 0.417 3 ==> 4

24 1 0.286 1 ==> 0

25 1 0.333 1 ==> 0

node_50 --> E. teilhardi 64 1 0.143 1 ==> 0

71 1 0.375 2 --> 1

72 1 0.375 2 ==> 1

119 1 0.300 0 --> 2

125 1 0.300 0 ==> 2

node_55 --> node_54 1 1 0.385 0 --> 5

2 1 0.500 1 ==> 2

4 1 0.333 0 ==> 1

5 1 0.667 1 ==> 2

7 1 0.417 3 --> 6

10 1 0.500 1 ==> 2

40 1 0.273 1 --> 0

42 1 0.500 0 --> 1

56 1 0.500 2 --> 1

70 1 1.000 2 ==> 1

78 3 0.333 3 --> 0

95 1 1.000 0 --> 1

100 1 0.500 1 --> 2

120 1 0.429 1 ==> 0

122 1 1.000 1 ==> 2

124 1 1.000 1 ==> 2

128 1 0.500 1 ==> 0

node_54 --> Hippidion saldia 24 1 0.286 1 ==> 2

Bootstrap method with heuristic search:

Number of bootstrap replicates = 1000

Starting seed = 1515151744

Optimality criterion = parsimony

Character-status summary:

Of 129 total characters:

24 characters are of type 'ord' (Wagner)

105 characters are of type 'unord'

All characters have equal weight

23 characters are constant

5 variable characters are parsimony-uninformative

Number of parsimony-informative characters = 101

Gaps are treated as "missing"

Starting tree(s) obtained via stepwise addition

Addition sequence: random

Number of replicates = 1000

Starting seed = 2069002862

Number of trees held at each step during stepwise addition = 1

Branch-swapping algorithm: tree-bisection-reconnection (TBR)

Steepest descent option not in effect

Initial 'MaxTrees' setting = 100

Branches collapsed (creating polytomies) if maximum branch length is zero

'MulTrees' option in effect

Topological constraints not enforced

Trees are unrooted

The limit of 100 trees (= 'MaxTrees') has been reached.

Do you want to increase 'MaxTrees'? (Y/n) paup> y;

Enter new value for 'MaxTrees' (200):paup> 200

Action if limit is hit:

(1) Prompt for new value

(2) Automatically increase by 100 (= AUTOINC)

(3) Leave unchanged, and don't prompt

(1)> paup> 2

1000 bootstrap replicates completed

Time used = 06:55:00.6

Bootstrap 50% majority-rule consensus tree

*/--------------------------------------------------------- Tapirus terrestris(1)*

*|*

*| /------- Hyrachyus eximius(2)*

*+-----------------------67------------------------+*

*| \------- Trigonias osborni(3)*

*|*

*| /------- Merychippus insignis(4)*

*| /--------------------90--------------------+*

*| | \------- Merychippus isoneus(5)*

*| |*

*| +-------------------------------------------------- Cormohipparion occidentale(6)*

*| |*

*| | /------------------------------------------- Pliohippus pernix(7)*

*| | |*

*| | | /------- Dinohippus leidyanus(8)*

*| | | /---------51----------+*

*| | | | \------- Dinohippus mexicanus(9)*

*\-100--+ | |*

*| | | /------- Equus simplicidens(12)*

*| | | /-----95------+*

*| | | | \------- E. qingyangensis(13)*

*| | | |*

*| | | +--------------------- E. eisenmannae(14)*

*| | | |*

*| | | +--------------------- E. sanmeniensis(15)*

*| | | |*

*| | /--52--+ +--------------------- E. huanghoensis(16)*

*\--69--+ | | |*

*| | | +--------------------- E. teilhardi(17)*

*| | | |*

*| | | +--------------------- E. livenzovensis(18)*

*| | | |*

*| | | +--------------------- E. stenonis(19)*

*| | | |*

*| | | +--------------------- E. senezensis(20)*

*| | | |*

*| | \--99---+ /------- E. sp. Dmanisi(21)*

*| | +-----77------+*

*| | | \------- E. oldowayensis (23)*

*| | |*

*| | +--------------------- E. koobiforensis(22)*

*| | |*

*\--56--+ +--------------------- E. grevyi(24)*

*| |*

*| | /-------------- E. quagga(25)*

*| | |*

*| +--57--+ /------- E. hemionus(27)*

*| | \--99--+*

*| | \------- E. kiang(28)*

*| |*

*| +--------------------- E. zebra(26)*

*| |*

*| | /------- E. przewalskii(29)*

*| \-----100-----+*

*| \------- E. ferus(30)*

*|*

*| /------- Hippidion principale(10)*

*\------------100-------------+*

*\------- Hippidion saldiasi(11)*

paup> upgma bionj=no showtree=yes breakties=random tieseed=382851232 brlens=yes;

UPGMA search settings:

Ties (if encountered) will be broken randomly; initial seed = 382851232

Distance measure = total character difference

(Tree is rooted)

Note: Ties were encountered; UPGMA tree may not be unique

Tree found by UPGMA method stored in tree buffer

Time used = 0.00 sec

Connected Branch Clustering

Node to node length level

-------------------------------------------------------------

59 (root) - 50.83732

40 59 4.71866 41.40000

35 40 1.42222 38.55555

32 35 11.77778 15.00000

31 32 1.50000 12.00000

Tapirus terrestr (1)* 31 6.00000 -

Hyrachyus eximiu (2)* 31 6.00000 -

Trigonias osborn (3)* 32 7.50000 -

34 35 2.52778 33.50000

33 34 13.75000 6.00000

Merychippus insi (4)* 33 3.00000 -

Merychippus ison (5) 33 3.00000 -

Cormohipparion o (6) 34 16.75000 -

39 40 4.20000 33.00000

37 39 7.25000 18.50000

Pliohippus perni (7) 37 9.25000 -

36 37 0.75000 17.00000

Dinohippus leidy (8) 36 8.50000 -

Dinohippus mexic (9) 36 8.50000 -

38 39 16.00000 1.00000

Hippidion princi (10) 38 0.50000 -

Hippidion saldia (11) 38 0.50000 -

58 59 10.13925 30.55882

56 58 2.12465 26.30952

53 56 1.13961 24.03030

42 53 1.51515 21.00000

41 42 10.00000 1.00000

Equus simplicide (12) 41 0.50000 -

E. qingyangensis (13) 41 0.50000 -

E. eisenmannae (14) 42 10.50000 -

52 53 1.49729 21.03572

48 52 2.01786 17.00000

47 48 1.70000 13.60000

46 47 0.13333 13.33333

43 46 2.16667 9.00000

E. sanmeniensis (15) 43 4.50000 -

E. huanghoensis (16) 43 4.50000 -

45 46 2.41667 8.50000

44 45 1.75000 5.00000

E. livenzovensis (18) 44 2.50000 -

E. stenonis (19) 44 2.50000 -

E. koobiforensis (22) 45 4.25000 -

E. teilhardi (17) 47 6.80000 -

E. senezensis (20) 48 8.50000 -

51 52 2.76786 15.50000

49 51 5.25000 5.00000

E. altidens Dman (21) 49 2.50000 -

E. oldowayensis (23) 49 2.50000 -

50 51 3.25000 9.00000

E. grevyi (24) 50 4.50000 -

E. zebra (26) 50 4.50000 -

55 56 3.65476 19.00000

E. quagga (25) 55 9.50000 -

54 55 8.50000 2.00000

E. hemionus (27) 54 1.00000 -

E. kiang (28) 54 1.00000 -

57 58 13.27941 4.00000

E. przewalskii (29) 57 2.00000 -

E. ferus (30) 57 2.00000 -

-------------------------------------------------------------

Sum 281.248

UPGMA tree:

/-------------- Tapirus terrestris

/--31

| \-------------- Hyrachyus eximius

/--------------------------32

| \------------------ Trigonias osborn

|

/--35 /------- Merychippus insignis

| | /-------------------------------33

| | | \------- Merychippus isoneus

| \----34

| \---------------------------------------- Cormohipparion occidentale

|

/---------40 /---------------------- Pliohippus pernix

| | |

| | /----------------37 /-------------------- Dinohippus leidyanus

| | | \36

| | | \-------------------- Dinohippus mexicanus

| \--------39

| | /- Hippidion principale

| \-------------------------------------38

| \- Hippidion saldiasi

|

| /- Equus simplicidens

| /----------------------41

| | \- E. qingyangensis

| /--42

| | \------------------------- E. eisenmannae

| |

| | /----------- E. sanmeniensis

| | /---43

| | | \----------- E. huanghoensis

59 | |

| | 46 /------ E. livenzovensis

| | | /--44

| /-53 | | \------ E. stenonis

| | | /--47----45

| | | | | \---------- E. koobiforensis

| | | | |

| | | /---48 \---------------- E. teilhardi

| | | | |

| | | | \-------------------- E. senezensis

| | | |

| | \--52 /------ E. altidens Dmanisi

| /---56 | /-----------49

| | | | | \------ E. oldowayensis

| | | \----51

| | | | /----------- E. grevyi

| | | \------50

| | | \----------- E. zebra

| | |

\----------------------58 | /----------------------- E. quagga

| | |

| \-------55 /-- E. hemionus

| \-------------------54

| \-- E. kiang

|

| /----- E. przewalskii

\------------------------------57

\----- E. ferus
