# Supplementary material for: Evolution of Old World Equus and origin of the zebra-ass clade
Source: Sci Rep. 2021 May 12;11:10156. doi: 10.1038/s41598-021-89440-9 (PMC8114910; doi:10.1038/s41598-021-89440-9)
Supplement: Supplementary file 2 — Supplementary Information 2. [file 41598_2021_89440_MOESM2_ESM.doc]

**Evolution of Old World *Equus* and origin of the zebra-ass clade.**

Omar Cirilli^1,2*^, Luca Pandolfi^2*^, Lorenzo Rook^2^, Raymond L. Bernor^3,4^

^1^ Dottorato di Ricerca in Scienze della Terra, Università di Pisa, Via S. Maria 53, 56126 Pisa, Italy; ^2^ Dipartimento di Scienze della Terra, Paleo[Fab]Lab, Università degli Studi di Firenze, Via G. La Pira 4, 50121 Firenze, Italy;

^3^ College of Medicine, Department of Anatomy, Laboratory of Evolutionary Biology, 520 W St. N.W., 20059, Washington D.C., USA;

^4^ Human Origins Program, Department of Anthropology, Smithsonian Institution, 20560, Washington D.C., USA;

*Corresponding author

**Supplementary information**

**Measurements and anatomical nomenclature for skull multivariate analyses**

Measurements were taken to the nearest 0.1 mm, following the international equid measurement guidelines^42,43^. Anatomical nomenclature and osteological landmarks follow the international equid measurement guidelines^43^. *Hippotherium primigenium* from the Late Miocene of Hoewenegg, Germany (10.3 Ma)^43^ has been used as standard in Log10 Ratios diagrams.

Log10 Ratio diagrams, PCA and boxplots on skulls include the following measurements, represented in Figure S1:

M1(basal length of the muzzle); M2 (palatal length); M3 (vomerine length); M4 (post vomerine length: from the middle of the vomerine notch to basion); M5 (post palatal length, from the base of the palatal spur up to basion); M6 (total basal length of the skull); M7 (upper premolar row length, P2 - P4); M8 (upper molar row length, M1 - M3); M9 (upper cheek tooth row length, P2 - M3); M23 (anterior ocular line: from the prosthion to the most external point of the orbital process); M30 (length of the naso-incisival notch: from the prosthion to the back of the narial opening); M31 (cheek length: from the back of the narial opening to the most anterior point of the orbit);

M35 (perpendicular maximal height; M36: distance between POF and the facial maxillary crest);

M38 (heigh of the back of the POF above the alveolar border).

We did not include M24 (posterior ocular line: from the most exterior point of the orbital process to the middle of the supra-occipital crest) due poor preservation of many skulls, which could mis-estimate the real morphometric value.

**
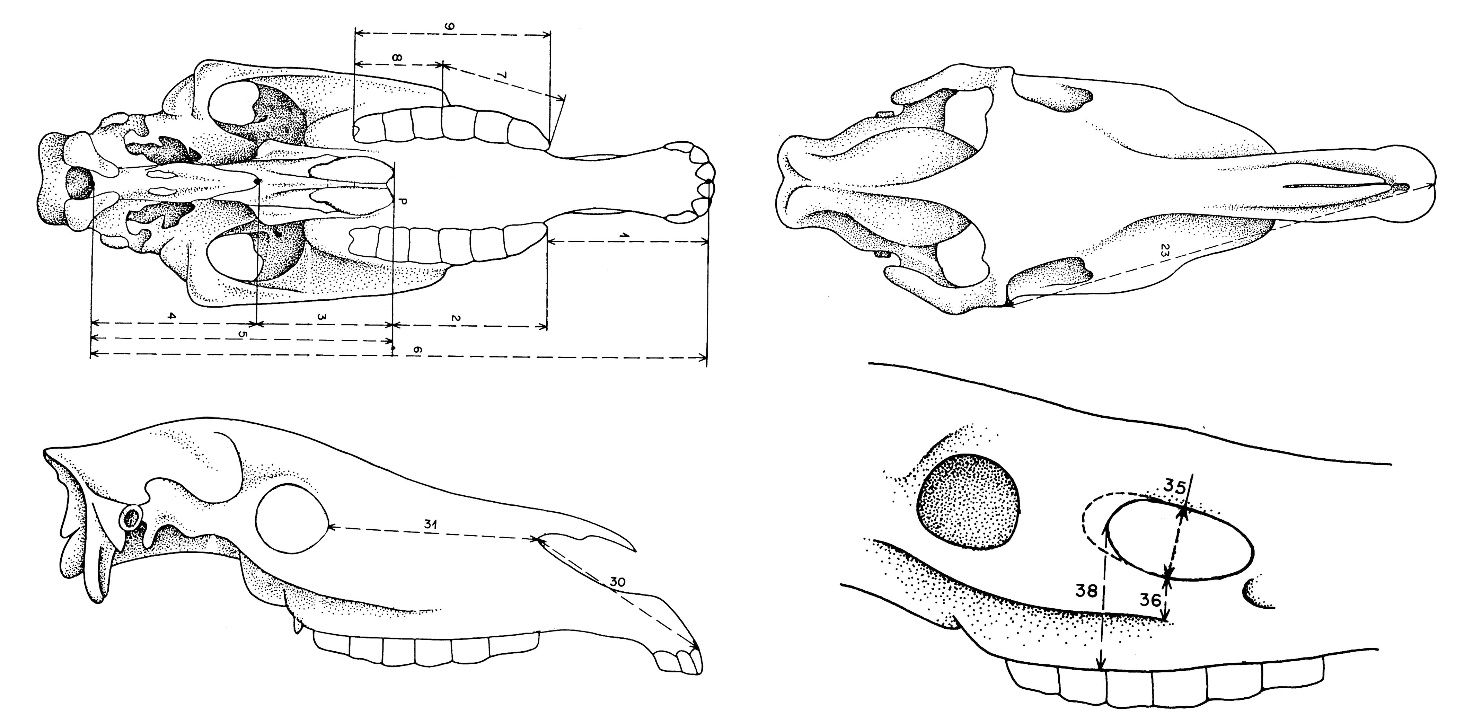
**

**Fig. S1.** Selected measurements used in the Log10 Ratio Diagrams and in the Principal Component Analysis. The measurements are referred to international equid measurements standards^48,49^.

**Morphometric analyses**

Figure S2 provides Log10 Ratios diagrams of *E. simplicidens*, *E. koobiforensis* and *E. grevyi* compared to Chinese (a) and European species (b). In both diagrams, the M1 is generally correlated with the total length of the skull (M6), whereas a different M3 - M4 pattern of the basal morphology of the skull can be observed. Compared to *E. koobiforensis* and *E.* *grevyi*, *E. simplicidens* and the Chinese species *E. eisenmannae*, *E. qingyangensis*, *E.* *huanghoensis* have a more elongated vomerine length (M3) and a reduced post vomerine length (M4), (Fig. S2a). *Equus sanmeniensis* and *E. yunnaniensis* exhibits longer values of M4, even if M3 is not reduced. Nevertheless, the European *E. stenonis*, represented by the holotype and the Saint Vallier sample, shows a basal skull morphology close to *E. koobiforensis* and *E. grevyi*, with the remarkable similar dimensions of vomerine length (M3) and post vomerine length (M4) (Fig. S2b). The dimensions of the upper toothrow remains conservative from *E. simplicidens* through the Old World *Equus* species and it is found in *E. grevyi* with larger M7 than M8 dimension, whereas the anterior ocular line (M23) is related with the basal length of the skull (M6), more elongated in larger skulls. The length of the naso-incisival notch (M30) and the cheek length (M31) exhibit an opposite development: when M30 is more elongated, M31 is more reduced, and vice-versa. *Equus stenonis* and the Early Pleistocene species *E. eisenmannae*, *E. sanmeniensis* and *E. koobiforensis* have the most elongated M30 values. Nevertheless, *E. simplicidens* and *E. grevyi* exhibit close values for M30 and M31, with M30 slight shortened in *E. grevyi*. The Log10 ratio pattern shown by *Equus* sp. from Senèze is almost identical to *E. stenonis*, even narrower in size.

**
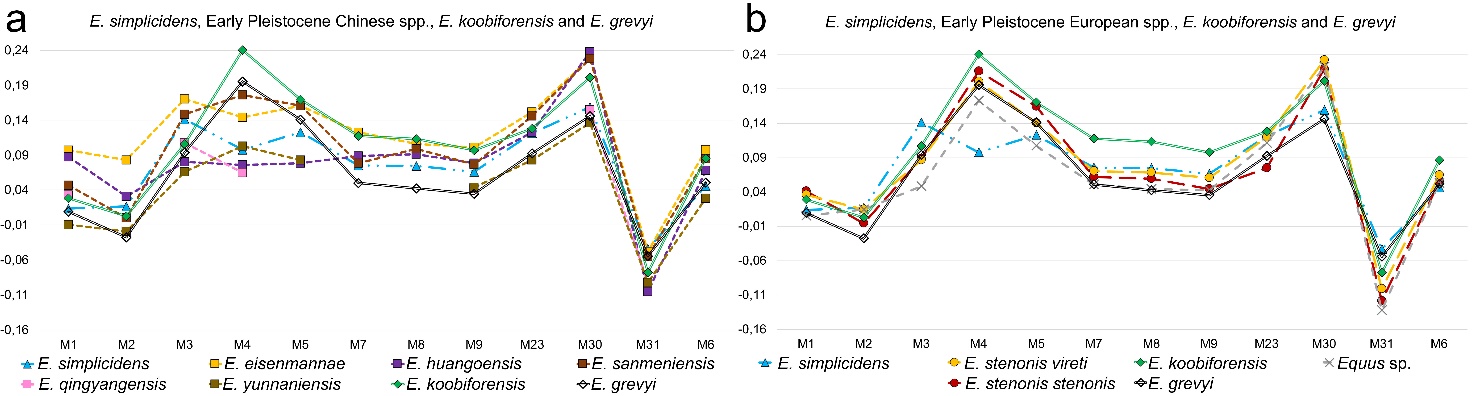
**

**Fig. S2.** Log10 Ratio diagrams on selected skull measurements. The Hoewenegg *Hippotherium primigenium*^49^ has been used as standard to develop the analyses.





**Fig. S3.** Loadings plots of the Principal Component Analyses based on the selected skull measurements (see Measurements and anatomical nomenclature for skull multivariate analyses). Letters are referred to the same plots shown in Figure 3. Plots primarily obtained by R Studio Software v.1.4.103 2020^50^ packages *ggplot2()* v.3.3.3^51^ and *prcomp()* v.3.6.2^52^.


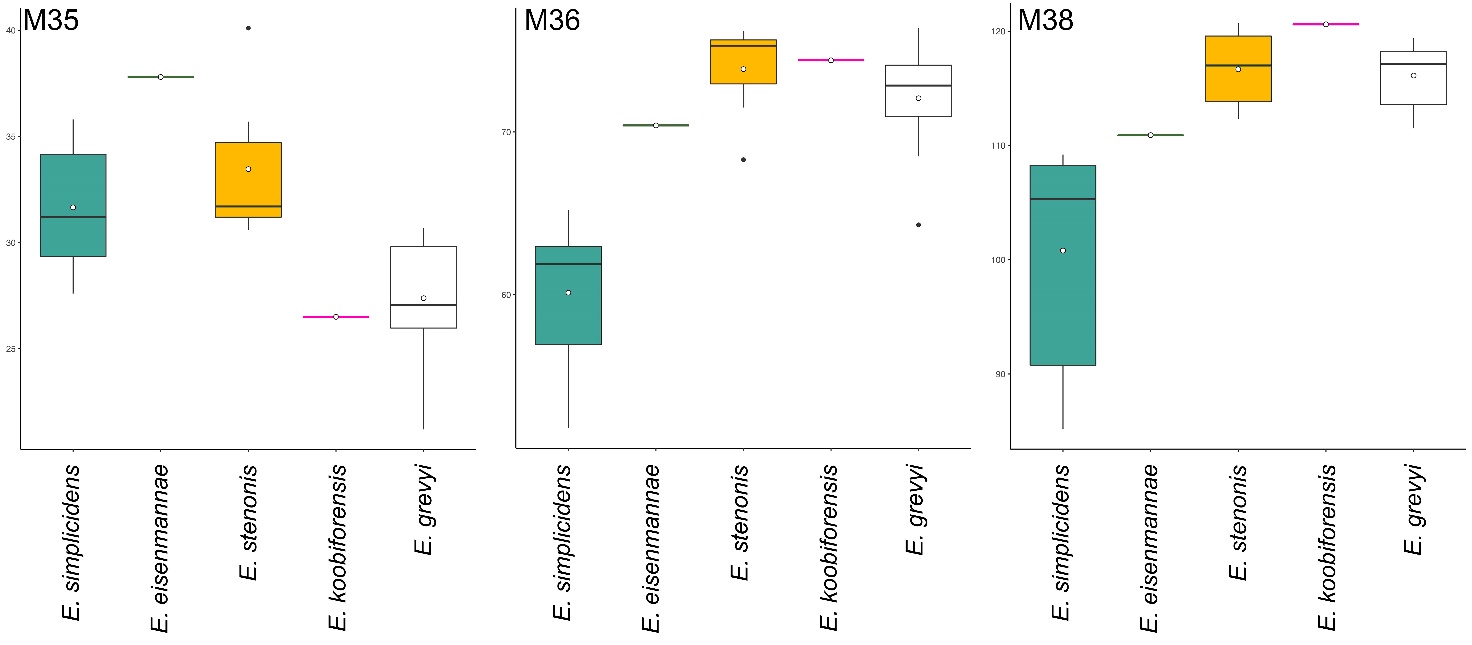


**Fig. S4.** Morphometrical comparison of the pre-orbital fossae (POF) by its perpendicular maximal height (M35), distance between POF and the facial maxillary crest (M36) and its height of the back of the POF above the alveolar border (M38). *Equus simplicidens*, *E. eisenmannae* and *E. stenonis* have greater dimensions of POF height (M35), whereas *E. koobiforensis* and *E. grevyi* show a more reduced M35. Furthermore, in *E. simplicidens* the POF is closer to the facial maxillary crest (M36) and the alveolar border (M38) if compared with *E. stenonis*, *E. koobiforensis* and *E. grevyi*. This evidence suggests that the POF underwent an evolutionary reduction with its restriction higher on the face being related to the increase in crown height of the maxillary cheek teeth. Plots primarily obtained by R Studio Software v.1.4.103 2020^50^, package *ggplot2()* v.3.3.3^51^


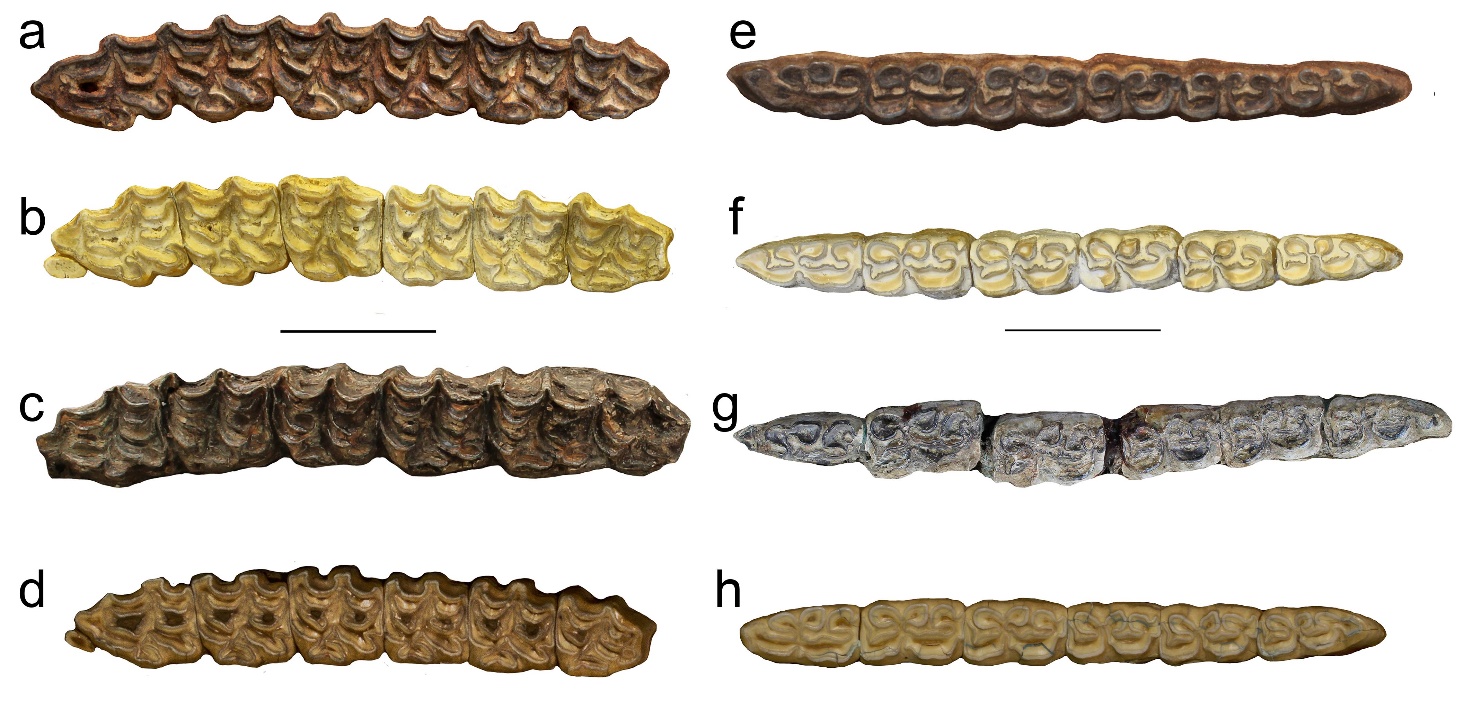


**Fig. S5.** Occlusal morphology of maxillary and mandibular cheek teeth rows of *E. simplicidens* (a, e; USNM12573), *E. stenonis* (b, f; NHML20.163355A), *E. koobiforensis* (c, g; KNM-ER1284) and *E. grevyi* (d; h USNM163228).

The figure shows the progressive evolution of the lingual margin of the protocone (Figs. a-d) from a shallow (a-b) to a deep depression (c-d), and the evolution of the metastylid shape from *E. simplicidens* to *E. grevyi.* (Figs. e-h). *Equus stenonis* (f) shows the same metaconid-metastylid shape as *E. koobiforensis* (g) and *E. grevyi* (h). Scale bar 5 cm.

USNM National Museum of Natural History, Washington D.C, USA.

NHML: Natural History Museum, Lyon, France;

KNM-ER: Kenyan Natural History Museum, East Rudolf, Nairobi (Kenya)

**List of species used in the Cladistic Analysis**

------------------------------------------------------------------------------------------------------------------------

*Tapirus terrestris*

**Data:** Personal observations; Antoine, 2002; Antoine et al., 2010.

**Specimens:** HNHM 77.299.1; MNHN no code; MSNF no code.

**HNHM**: Hungarian Natural History Museum, Budapest, Hungary.

**MNHN:** Muséum National d’Historie Naturelle; Paris, France.

**MSNF**: Museo di Sotira Naturale, Università di Firenze, sezione di Zoologia “La Specola”, Firenze, Italy.

------------------------------------------------------------------------------------------------------------------------

*Hyrachyus eximius*

**Data:** Personal observations; Antoine, 2002; Antoine et al., 2010.

**Specimens:** AMNH 5065; AMNH 12354; AMNH 12355; AMNH 12364; AMNH 107978; AMNH 12371.

**AMNH**: American Museum of Natural History, New York, USA.

------------------------------------------------------------------------------------------------------------------------

*Trigonias osborni*

**Data:** Personal observations; Antoine, 2002; Prothero, 2005; Antoine et al., 2010; Pandolfi, 2015.

**Specimens:** AMNH 9847; CMNH 97; CMNH 897; CMNH 8929; DMNH 890; DMNH 1056; DMNH 1510; DMNH 24800; MSM 97-21

**AMNH**: American Museum of Natural History, New York, USA.

**CMNH**: Carnegie Museum of Natural Histort, Pittsburgh, USA.

**DMNH**: Denver Museum of Natural History, Denver, USA.

**MSM**: Mesa Southwest Museum, USA.

------------------------------------------------------------------------------------------------------------------------

*Merychippus insignis*

**Data:** Vera Eisenmann Website <https://vera-eisenmann.com/> ; Woodbourne, M.O., 2003;

**Specimens:** FAM 870001; FAM 87003; FAM 87001; FAM 87004; FAM 87005; FAM 87072; FAM 87078; FAM 87080; ANSP 11276; FAM. No 87006; FAM No. 87000; FAM 87002;

**FAM:** Frick Collection, American Museum of Natural History; New York, USA.

**ANSP:** Academy of Natural Science of Philadelphia, USA.

------------------------------------------------------------------------------------------------------------------------

*Merychippus isoneus*

**Data:** Personal observations.

**Specimens:** AMNH 14179, complete skeleton (cast housed in the Natural History museum in Florence)

**AMNH:** American Museum of Natural History; New York, USA.

------------------------------------------------------------------------------------------------------------------------

*Cormohipparion occidentale*

**Data:** Personal observations; MacFadden, 1984; Bernor et al., 2018;

**Specimens:** FAM71800; FAM71855; FAM71856; FAM71857; FAM71858; FAM71859; FAM71860; FAM71861; FAM71862; FAM71863;

**FAM:** Frick Collection, American Museum of Natural History; New York, USA.

------------------------------------------------------------------------------------------------------------------------

*Pliohippus pernix*

**Data:** Personal observations.

**Specimens:** YPM13007; partial skeleton.

**YPM:** Yale Peabody Museum, New Haven, USA.

------------------------------------------------------------------------------------------------------------------------

*Dinohippus leidyanus*

**Data:** Personal observations; Azzaroli 1986;

**Specimens:** AMNH 18972; AMNH 87201; AMNH 116129; AMNH 116970; AMNH116134; AMNH 116135; AMNH 87204; AMNH 87208; AMNH 87209; AMNH 87112;

**AMNH:** American Museum of Natural History; New York, USA.

------------------------------------------------------------------------------------------------------------------------

*Dinohippus mexicanus*

**Data:** Personal observations; MacFadden, B., and Carranza-Castañada, 2002

**Specimens:** IGM 7596; MPGJ 1982; MPGJ 1683; MPGJ 103; MPGJ 5132; MPGJ 1967; IGCU 11503; MPGJ 3987; MPGJ 4040; MPGJ 1982; IGM 7596; MPGJ 856; MPGJ 5129; MPGJ 5126; MPGJ 857; MPGJ 1683

**IGM:** Instituto de Geología; Juriquilla, Mexico

**IGCU:** Instituto de Geología, Ciudad Universitaria; Juriquilla, Mexico

**MPGJ:** Museo de Paleontología Geociencias; Juriquilla, Mexico

------------------------------------------------------------------------------------------------------------------------

*Hippidion principale*

**Data:** Personal observations; MacFadden, 1997; Alberdi et al., 1996; Alberdi et al., 2001;

**Specimens:** Complete skeleton, fig. 2 in MacFadden, 1997; FMNH14200; MPH-P067; MMP-2600.

**FMNH:** Vertebrate Paleonotology (Fossil Mammal) Field Museum of Natural History; Chicago, USA.

**MMP:** Museo de Ciencias ‘Lorenzo Scaglia,’ Mar Del Plata, Buenos Aires, Argentina

**MPH:** Museo Municipal ‘Punta Hermengo,’ Miramar, Argentina

------------------------------------------------------------------------------------------------------------------------

*Hippidion saldiasi*

**Data:** Personal observations; Alberdi et al., 2007; Alberdi et al., 2001

**Specimens:** Betecsa 1, AEP1 F(e) 195/1

------------------------------------------------------------------------------------------------------------------------

*Equus simplicidens*

**Data:** Personal observations.

**Specimens:** USNM11989; USNM12481; USNM11989; USNM12501; USNM12522; USNM12528; USNM12535; USNM12538; USNM12542; USNM12543; USNM12546; USNM12547; USNM12548; USNM12573; USNM12576; USNM13793; USNM13835; USNM13841; USNM13841; USNM13842; USNM16785; USNM16982; USNM16985; USNM16987; USNM16995; USNM12522; USNM12538; USNM12546; USNM12570; USNM13791; USNM13792; USNM12793; USNM17000; USNM 16320; USNM 13791; USNM 12573; USNM222034; USNM222041; USNM222025; USNM222036; USNM222029; USNM 13791; USNM 12573; USNM 16320; USNM 13791; USNM 12573; USNM 16320; USNM 13791; USNM 12573

**USNM:** National Museum of Natural History, Washington D.C, USA.

------------------------------------------------------------------------------------------------------------------------

*Equus qingyangensis*

**Data:** Personal observations; Deng, 1999; Sun et al., 2017; Sun et al., 2020

**Specimens:** NWUV1128; NWUV1129; NWUV1134; NWUV1152; NWUV1155; NWUV1128; NWUV DLJ-2016-11-09; NWUV - M 1324

**NWUV:** Vertebrate Paleontology of Institute of Cenozoic Geology and Environment, Northwest University; Xian, China.

**DLJ:** Dalian Jinyuan Cave, China.

------------------------------------------------------------------------------------------------------------------------

*Equus eisenmannae*

**Data:** Personal observations; Qiu et al., 2004 ; Sun and Deng, 2019;

**Specimens:** IVPP V 13552; HMV1V 1105; HMV 1103; HMV 1105; LDU008; LDL009; LDL012; HZT022; HZT023; HZT024; HZT025; HZT026; HZT027; LDL032; LDL033; LDU013; LDU014; LDL015; LDL016; LDL017; LDL018; LDL019; LDL020; LDL022; HZT028; HZT029; HZU030; HZT031; HZU033; HZT034; HZT035; HZT037; HZT038; LDL035; LDL036; LDL037; LDL038;

**IVPP:** Institute of Vertebrate Paleontology and Paleoanthropology; Beijing, China.

**HPM**, Hezheng Paleozoological Museum, Hezheng, China;

------------------------------------------------------------------------------------------------------------------------

*Equus sanmeniensis*

**Data:** Personal observations; Kalmiakov, 2015; Sun and Deng, 2019;

**Specimens:** NIH 002;

**NIH:** Muséum National d’Historie Naturelle, Nihewan collection; Paris, France.

------------------------------------------------------------------------------------------------------------------------

*Equus huanghoensis*

**Data:** Personal observations;

**Specimens:** NWUV1403.1, NWUV1403.2; NWUV19039;

**NWUV:** Vertebrate Paleontology of Institute of Cenozoic Geology and Environment, Northwest University; Xian, China.

------------------------------------------------------------------------------------------------------------------------

*Equus teilhardi*

**Data:** Personal observations; Eisenmann, 1975; Sun et al., 2017;

**Specimens:** M 1321

**PMU M:** Museum of Evolution of Uppsala University; Uppsala, Sweden.

------------------------------------------------------------------------------------------------------------------------

*Equus livenzovensis*

**Data:** Personal observations, Azzaroli, 2000

**Specimens:** L4; L95; L229; L739; L779; RGU335; RGU570; L291; L860; L1278; RGU11; L911; UCBL-FSL 21173; IGF11074; IGF11075; IGF11224; IGF11225; IGF11226; IGF11236; IGF11275; IGF11276; IGF11277; IGF11282; IGF11285; IGF14689; IGF14690; IGF15371; IGF4093V; IGF954V.

**L:** Liventsovka, Regional Ethnographic Museum; Rostov – on – Don, Russia.

**RGU:** State University of Rostov; Rostov – on – Don, Russia.

**IGF:** Museo di Storia Naturale, Università di Firenze, Sezione Geologia e Paleontologia; Firenze, Italy.

------------------------------------------------------------------------------------------------------------------------

*Equus stenonis*

**Data:** Personal observations.

**Specimens:** IGF 560; IGF 530; IGF 538; IGF 560; IGF 583; IGF11024; IGF515; IGF 10336; IGF 11039; IGF 11040; IGF 11041; IGF 11044; IGF 11045; IGF 11051; IGF 11057; IGF 11066; IGF 11073; IGF 11314; IGF 11315; IGF 12824; IGF 12825; IGF 12826; IGF 12827; IGF 12828; IGF 12829; IGF 12830; IGF 12831; IGF 1818V; IGF 543; IGF 568; IGF 568; IGF 683/2; IGF 7558V; IGF 7559V; IGF 7608V; IGF11222; IGF11223; IGF11272; IGF11274; IGF11291; IGF11292; IGF11293; IGF4045V; IGF4046V; IGF4047V; IGF 11001; IGF 11005; IGF 11011; IGF 11013; IGF 11014; IGF 11033; IGF 11036; IGF 11037; IGF 11316; IGF 12833; IGF 12834; IGF 12835; IGF 12836; IGF 12837; IGF 1314V; IGF 1315V; IGF 1316V; IGF 13732; IGF 13734; IGF 13735; IGF 14209; IGF 14226; IGF 14227; IGF 15231; IGF 15232; IGF 1817V; IGF 4885V; IGF 544; IGF 548; IGF I567; IGF 569; IGF 7607V; IGF 7715V; IGF 7932V; IGF 8097V; IGF11226; IGF11263; IGF11264; IGF11265; IGF11267; IGF11268; IGF11269; IGF11270; IGF11271; IGF11287; IGF11288; IGF11289; IGF11290; IGF 14232; IGF 523; IGF 535; IGF 537; IGF 564; IGF 583; IGF11023; IGF11025; IGF562; NHML 20.163361; NHML 20.163735; NHML 20.163772; NHML 20.163362; NHML 20.163362b; NHML 20.163464; NHML 20.163464b; NHML 20.163741; NHML 20.163758; NHML 20.163757; NHML 20.163761; NHML 20.163770; NHML 20.163371; NHML 20.163972; NHML 20.163964; NHML 20.163968; NHML 20.163972; NHML 20.163975; NHML 20.163976; NHML 20.163981; NHML 20.163978; NHML 20.163481; NHML 20.163485; NHML 20.163480; NHML 20.163482; NHML 20.163483; NHML 20.163484; NHML 20.163421; NHML 20.163422; NHML 20.163423; NHML 20.163425; NHML 20.163419; NHML 20.163418; NHML 20.163412; NHML 20.163417; NHML 20.163420; NHML 20.163416; NHML 20.163438; NHML 20.163439; NHML 20.163491; NHML 20.163985; NHML 20.163986; NHML 20.163987; NHML 20.163988; NHML 20.163982; NHML 20.163983; NHML 20.163990; NHML 20.163984; NHML 20.163991; NHML 20.163970; NHML 20.163971; NHML 20.163962; NHML 20.163967; NHML 20.163969; NHML 20.163958; NHML 20.163955; NHML 20.163954; NHML 20.163490; NHML 20.163488; NHML 20.163492; NHML 20.163493; NHML 20.163489; NHML 20.163487; NHML 20.163486; NHML 20.163437; NHML 20.163436; NHML 20.163440; NHML 20.163354; NHML 20.163355; NHML 20.163358; NHML 20.163356; NHML 20.163360; NHML 20.163372; NHML 20.163724; NHML 20.163731; NHML 20.163364; NHML 20.163463;

**IGF:** Museo di Storia Naturale, Università di Firenze, Sezione Geologia e Paleontologia; Firenze, Italy.

**NHML:** Natural History Museum of Lyon; Lyon, France.

------------------------------------------------------------------------------------------------------------------------

*Equus senezensis*

**Data:** Personal observations.

**Specimens:** NHML SZ30; NHML SZ2; NHML SZ38; NHML SZ5; NHML SZ24; UCBL-FSL 210832; UCBL-FSL 210842; UCBL-FSL 210835; UCBL-FSL 210843; UCBL-FSL 210841; UCBL-FSL 210834; UCBL-FSL 210844; UCBL-FSL 210845; UCBL-FSL 210842; UCBL-FSL 210849; UCBL-FSL 210850; UCBL-FSL 210851; UCBL-FSL 210859; UCBL-FSL 210857; UCBL-FSL 210895; UCBL-FSL 210886; UCBL-FSL 210884; UCBL-FSL 210890; UCBL-FSL 210887; UCBL-FSL 210888; UCBL-FSL 210889; UCBL-FSL 210882; UCBL-FSL 210883; UCBL-FSL 210917; UCBL-FSL 210920; UCBL-FSL 210897; UCBL-FSL 210921; UCBL-FSL 210922; UCBL-FSL 546597; UCBL-FSL 546610; UCBL-FSL 546647; UCBL-FSL 546655; UCBL-FSL 546673; UCBL-FSL 1116

**NHML:** Natural History Museum of Lyon; Lyon, France.

**UCBL-FSL:** Université Claude Bernard-1, Paleontological Collection; Lyon, France;

------------------------------------------------------------------------------------------------------------------------

*Equus* sp. Dmanisi

**Data:** Personal observations.

**Specimens:** D2589; D2590; D3697; Dm55/61.1.B1g1.111; D2471; D2620; D3870; D4691; D613; D614; D1350; D1591; D2261; D22618; D2309; D3060; D3152; D3379; D4390; D4864; D529; D5365; D5705; D6851; D717e2; D793; D887; Dm1088; Dm159; Dm50/63i-106; Dm53/59.3.B1g1.178; Dm53/59.3.B1g1.183; Dm61; Dm62; Dm62/63B2a12133; Dm64/64.3B1X48 5620; Dm65/61 VI 43 3066; Dm69/61.2.B1K.67; Dm70/34.3A4.45; Dm8/151.1.A4.26; D2034; D2501; Dm60/5 1I 20; Dm3V246; Dm53/60.3B.Gl.89; Dm64/60Va3918; D3065; D1973; D1838; D1586; D3953; Dm62/62.4B14 174 5640; Dm64/62.381X243 5372; D1235; D1586; D2172; D2172; D2239; D2298; D2311; D2328; D2807; D3344; D335; D3988; D3988; D3993; D3993; D4758; D5200; D5364; D5640; D68; D709; D71; D886; Dm2281; Dm50/62 IV5756 2305; Dm64/63.381x479; Dm64/63.3B1x479 5364; Dm64/63.3blk5407 5144; Dm64/64.1.B1Z.157; Dm65/70.VI.107; Dm66/60 V 162 2622; Dm67/61; Dm67/61 IV 47 3344; Dm68/61 IV 26 2810; Dm7/150.3.B.1; Dm709; Dm71; Dm812; Dm89; DmM6/5.B2.43; D353; D66; Dm53/59.3B1g1.192;

**D:** Old Dmanisi Collection, S. Janashia Museum of Georgia, Georgian National Museum; Tbilisi, Republic of Georgia.

**Dm:** New Dmanisi Collection, S. Janashia Museum of Georgia, Georgian National Museum; Tbilisi, Republic of Georgia.

------------------------------------------------------------------------------------------------------------------------

*Equus koobiforensis*

**Data:** Personal observations. Eisenmann, 1983.

**Specimens:** KNM-ER1484; KNM-ER5519; KNM-ER5361; KNM-ER2691; KNM-ER2687; KNM-ER4025; KNM-ER1129; KNM-ER1255; KNM-ER2062; KNM-ER2688; KNM-ER1281; KNM-ER1226; KNM-ER1258; KNM-ER1241; KNM-ER1268; KNM-ER5360; ; KNM-ER333; ; KNM-ER4026; ; KNM-ER3986; KNM-ER4015; ; KNM-ER4027; ; KNM-ER4046; ; KNM-ER1275; KNM-ER1276; ; KNM-ER5358; ; KNM-ER4052; ; KNM-ER1275.

**KNM – ER:** Kenyan Natural History Museum, East Rudolf; Nairobi, Kenya.

------------------------------------------------------------------------------------------------------------------------

*Equus oldowayensis* (Olorgesaile)

**Data:** Personal observations, Bernor et al., 2019; Churcher and Hooijer, 1980.

**Specimens:** KNM – OG 22833; RMNH 490-4; RMNH 67-129; RMNH 740-25; RMNH L.513-13; RMNH 627-95; RMNH 508-1a; RMNH 48-21; RMNH 627-366; RMNH 626-87; RMNH 627-211; RMNH K4; RMNH F17-7; RMNH F17-10; RMNH F17-14; RMNH F17-9; RMNH F17-6; RMNH F17-8; RMNH F17-5; RMNH F32-1; RMNH F23-3; RMNH P.994-5; RMNH P.996-39; RMNH P.996-7; RMNH P.1000-6; RMNH OMO1967(65); RMNH F513-34; RMNH F410-5; RMNH F356-4; RMNH 7-3; RMNH 7-4;

**KNM – OG:** Kenyan Natural History Museum, Olorgesaile; Nairobi, Kenya.

**RMNH:** Rijksmuseum van Natuurlijke Historie; Leiden, Netherlands.

------------------------------------------------------------------------------------------------------------------------

*Equus grevyi*

**Data:** Personal observations.

**Specimens:** USNM152231; USNM161927; USNM162963; USNM163228; USNM163331; USNM163332; USNM163333; USNM163334; USNM163338; USNM173041; USNM182026; USNM182027; USNM182028; USNM182063; USNM199079; USNM241009; USNM49796; USNM49944.

**USNM:** National Museum of Natural History, Washington D.C, USA.

------------------------------------------------------------------------------------------------------------------------

*Equus quagga*

**Data:** Personal observations.

**Specimens:** USNM015120; USNM061743; USNM061744; USNM269165; USNMA22977; USNM161928; USNM162950; USNM162951; USNM162952; USNM5342228; USNM534228; USNM534228B; USNM534828; USNM61743; USNMA22870; USNMA22977; USNM161927; USNM162954; USNM162955; USNM162959; USNM162960; USNM162961; USNM162962; USNM162954; USNM163235; USNM163236; USNM163237; USNM163239; USNM164520; USNM164632; USNM181840; USNM252096; USNM259848; USNM38211; USNM38212;

**USNM:** National Museum of Natural History, Washington D.C, USA.

------------------------------------------------------------------------------------------------------------------------

*Equus zebra*

**Data:** Personal observations; Vera Eisenmann Website <https://vera-eisenmann.com/>

**Specimens:** USNM197983; USNM270125; USNM270514; AMNH83602; AMNH7691;

**USNM:** National Museum of Natural History, Washington D.C, USA.

**AMNH:** American Museum of Natural History; New York, USA.

------------------------------------------------------------------------------------------------------------------------

*Equus hemionus*

**Data:** Personal observations; Vera Eisenmann Website <https://vera-eisenmann.com/>

**Specimens:** USNM327091; USNM521103; USNM541427; USNM581897; USNM581994; MCZ6345; MNHN383; LG19046; LG27139;

**USNM:** National Museum of Natural History, Washington D.C, USA.

**MCZ:** Harvard University; Cambridge, USA.

**MNHN:** Muséum National d’Historie Naturelle; Paris, France.

**ZIN:** Zoological Institute, Saint Petersburg, Russia.

------------------------------------------------------------------------------------------------------------------------

*Equus kiang*

**Data:** Personal observations; Vera Eisenmann Website <https://vera-eisenmann.com/>

**Specimens:** USNM084081; USNM084088; USNM49493; USNM49796; USNM84083; USNM84088; USNM49493; USNM304614; NHMUK1939.2472; NHMUK1879.11.21.182; NHMUK1905.6.20.1; NHMUK1894.2.8.3; NHMUK1860.4.5.77; NHMUK1851.7.16.4; NHMUK1848.6.11.17; NHMUK1848.6.11.6; NHMUK976;

**USNM:** National Museum of Natural History, Washington D.C, USA.

**NHMUK:** Natural History Museum; London, United Kingdom.

------------------------------------------------------------------------------------------------------------------------

*Equus przewalskii*

**Data:** Personal observations; Vera Eisenmann Website <https://vera-eisenmann.com/>

**Specimens:** AMNH90198; AMNH16234; NHMUK1945.6.11.1; NHMUK1907.5.15.1; AC1926-228; AC1941-332; AC1935-486; AC1932-46; MGU1772; ZIN27089; ZIN27031; ZIN5218; ZIN5214; ZIN5213; ZIN5212; MCZ5108; AMNH21523; AMNH32686; AMNH16234; MA1977.55; ZU10800; ZU10801; NHMUK1960.2.1.4; NHMUK1902.9.25.1; NHMUK1963.1.25.1; NHMUK1961.5.10.2; AC1896-269; ZIN57/29; MGU1772;

**AMNH:** American Museum of Natural History; New York, USA.

**NHMUK:** Natural History Museum; London, United Kingdom.

**ZIN:** Zoological Institute, Saint Petersburg, Russia

**AC:** Laboratoires d’Anatomie Comparée et des Mammifères du Muséum National d’Historie Naturelle; Paris, France.

**ZU:** Zoologisches Museum der Universität; Zürich, Switzerland.

------------------------------------------------------------------------------------------------------------------------

*Equus ferus*

**Data:** Personal observations; Vera Eisenmann Website <https://vera-eisenmann.com/>

**Specimens:** USNM399046; ZIN521; AMNH204174; AMNH204210;

**IGF:** Museo di Storia Naturale, Università di Firenze, Sezione Geologia e Paleontologia; Firenze, Italy.

**USNM:** National Museum of Natural History, Washington D.C, USA.

**AMNH:** American Museum of Natural History; New York, USA.

**ZIN:** Zoological Institute; Saint Petersburg, Russia

**SUPPLEMENTARY TABLE LEGENDS**

Table S1. Data matrix of the selected species used in the cladistic analysis.

Table S2. Components of the Principal Component Analysis (PCA) of equid skulls.

Tables S3. Skull dataset of the Principal Component Analysis (PCA) developed on the fossil Plio-Pleistocene species with the extant *Equus grevyi*.
